# High-Throughput Sequencing of microRNAs in Peripheral Blood Mononuclear Cells: Identification of Potential Weight Loss Biomarkers

**DOI:** 10.1371/journal.pone.0054319

**Published:** 2013-01-15

**Authors:** Fermín I. Milagro, Jonatan Miranda, María P. Portillo, Alfredo Fernandez-Quintela, Javier Campión, J. Alfredo Martínez

**Affiliations:** 1 Department of Nutrition, Food Sciences and Physiology, University of Navarra, Pamplona, Spain; 2 Department of Nutrition and Food Sciences, University of the Basque Country (UPV/EHU), Vitoria, Spain; Sapporo Medical University, Japan

## Abstract

**Introduction:**

MicroRNAs (miRNAs) are being increasingly studied in relation to energy metabolism and body composition homeostasis. Indeed, the quantitative analysis of miRNAs expression in different adiposity conditions may contribute to understand the intimate mechanisms participating in body weight control and to find new biomarkers with diagnostic or prognostic value in obesity management.

**Objective:**

The aim of this study was the search for miRNAs in blood cells whose expression could be used as prognostic biomarkers of weight loss.

**Methods:**

Ten Caucasian obese women were selected among the participants in a weight-loss trial that consisted in following an energy-restricted treatment. Weight loss was considered unsuccessful when <5% of initial body weight (non-responders) and successful when >5% (responders). At baseline, total miRNA isolated from peripheral blood mononuclear cells (PBMC) was sequenced with SOLiD v4. The miRNA sequencing data were validated by RT-PCR.

**Results:**

Differential baseline expression of several miRNAs was found between responders and non-responders. Two miRNAs were up-regulated in the non-responder group (mir-935 and mir-4772) and three others were down-regulated (mir-223, mir-224 and mir-376b). Both mir-935 and mir-4772 showed relevant associations with the magnitude of weight loss, although the expression of other transcripts (mir-874, mir-199b, mir-766, mir-589 and mir-148b) also correlated with weight loss.

**Conclusions:**

This research addresses the use of high-throughput sequencing technologies in the search for miRNA expression biomarkers in obesity, by determining the miRNA transcriptome of PBMC. Basal expression of different miRNAs, particularly mir-935 and mir-4772, could be prognostic biomarkers and may forecast the response to a hypocaloric diet.

## Introduction

The discovery and characterization of small non-coding molecules of RNA with approximately 20–30 nucleotides has radically contributed to reinterpret the function of RNA and the regulation of gene expression [1]. MicroRNAs (miRNAs) can modulate (generally inhibiting) the expression of over one-third of protein-coding genes by complementary binding to specific nucleotide regions of mRNAs [2]. In this sense, in the version 19 of miRBase database (http://www.mirbase.org), 25,141 mature miRNAs have been identified in 193 species, and each one can specifically target hundreds of genes (about 60% of mammalian genes), being their dysregulation putatively involved in the pathogenesis of many chronic diseases including cancer, cardiovascular disease, type 2 diabetes and obesity [3].

Regarding obesity, miRNAs seem to participate in the regulation of several important biological processes, such as adipocyte differentiation, metabolic integration, fat metabolism and insulin sensitivity [4]. Thus, different investigations have compared the miRNA expression patterns between the undifferentiated preadipocytes and the differentiated adipocytes, both in 3T3-L1 murine cells [5] and in human subcutaneous preadipocytes [6]. These studies suggest that the miRNA expression patterns play an important role in the regulation of cell differentiation during the adipogenic process, accelerating or inhibiting the process, and hence regulating fat cell development. However, as occurs with many adipogenic genes whose expression increases during adipogenesis but decreases in adipocytes from *ob/ob* and diet-induced obese mice [7], many miRNAs show inverse regulatory patterns during adipogenesis and in obese patients [5]. These outcomes have been explained by the interference of hypoxia, macrophage infiltration and proinflammatory cytokines like TNF-α in the process [8]. Also, the miRNA expression patterns of omental and subcutaneous adipose tissue show some disparities that may contribute to intrinsic differences between both depots and that eventually could influence the development of some of the comorbidities associated to obesity [9].

The recent advances in sequencing and omics technologies have enabled the characterization of all the miRNAs that are expressed in a tissue or cell culture [10], allowing to identify new biomarkers of diagnosis and prognosis, but also to search for novel gene regulators for a number of chronic conditions, including obesity. In this context, the search for obesity-related biomarkers or prognostic markers of weight loss has been until now focused on genetics (SNP identification) or transcriptomics (gene expression), being whole blood or white cells the preferred biological sources because of the less invasive method of extraction. Thus, several SNPs have been identified in relation to the success of a weight loss program [11], whereas mRNA microarrays have been also used to early differentiate the populations of high and low responders to a hypocaloric diet [12]. In relation to epigenetics, which usually encompasses mitotically heritable but potentially reversible changes in DNA methylation, histone modifications and miRNA expression [13], most of the existing research has been focused on DNA methylation, studying the differences between obese and non-obese rodents [14]–[17] and humans [18], [19], but also between high and low responders to a low calorie diet [20]–[23] trying to search for epigenetic biomarkers of response to the diet. Concerning miRNAs, another epigenetic-related mechanism of gene expression regulation that is influenced by dietary manipulation [24], some studies have compared the expression differences between obese and non-obese individuals in both mice [25]–[27] and humans [6], [28], demonstrating that some miRNAs are altered in adipose tissue and circulation in obesity. Variations of miRNA expression seem also to be involved in the severity and susceptibility to nonalcoholic steatohepatitis (NASH) [29]. However, no studies have been focused on the early prediction of the weight loss outcome after a dietary treatment, which can be of great interest in the design of personalized nutrition.

The aim of the present research is to characterize the miRNA expression pattern of subjects with a different outcome to a hypocaloric dietary treatment by high-throughput sequencing in peripheral blood mononuclear cells (PBMC), in order to find prognostic biomarkers of response to the diet.

## Subjects and Methods

### Study Population and Ethics Statement

Ten Caucasian women with excess body weight (BMI = 35.6 kg/m^2^) were selected among the participants in a weight-loss trial that consisted in following an energy-restricted treatment according to a food exchange system (8-week low-calorie diet providing 800–880 kcal/day), as reported elsewhere [20]. The diet supplied 15% of energy as proteins, 55% as carbohydrates and 30% as fat [20]. After a detailed explanation of the study, a written informed acceptance was obtained from all participants in agreement with the Helsinki Declaration. This consent document and the study protocol were previously approved by the Ethical Committee of the Clínica Universidad de Navarra (Ref.090/2008). At the baseline, a screening including physical examination, medical history, activity patterns and fasting blood profile was performed. Body weight was recorded with a digital balance accurate to 0.1 kg (Seca 767, Vogel & Halke, Hamburg, Germany), and height was measured with a calibrated stadiometer (Seca 220, Vogel & Halke). Measurements were carried out in underwear after an overnight fast. Waist circumference was recorded at the midpoint between the superior iliac crest and lower costal margin whereas hip circumference was measured at the maximum protuberance of the buttocks. Body mass index (BMI) was calculated as body mass (kg) divided by height (m) squared. Total fat mass was measured by bioelectrical impedance with a Quadscan 4000 equipment (Bodystat, UK). Baseline circulating glucose was quantified by a specific colorimetric assay (Horiba ABX Diagnostics, Montpellier, France) using an automatized system (COBAS MIRA, Roche, Basel, Switzerland). Blood pressure was recorded with a standard mercury sphygmomanometer (Minimus II, Riester, Junginger, Germany) after at least 5 minutes of rest in a sitting position, following standardized procedures according to WHO criteria. Leisure time physical activity was estimated in MET hours by a validated questionnaire [30], and the number of hours devoted to different activities was multiplied by the specific MET score of each activity.

Weight loss was considered unsuccessful when it was ≤5% of initial body weight and successful when it was ≥5%. Based on this, the sample was categorized as unsuccessful (n = 5) or successful (n = 5) weight-loss subjects.

### miRNA Sequencing by SOLiD v4

At baseline, PBMCs were isolated by differential centrifugation using Polymorphprep (Axis Shield PoC AS, Oslo, Norway). The PBMC band was harvested with a Pasteur pipette and, after washing with phosphate-buffered saline (PBS), total RNA was extracted by using a commercial kit (mirVana, Ambion, Austin, TX, USA). RNA quality was assessed by measuring 1 µl of 100 ng/µl total RNA in a 2100 Bioanalyzer (Agilent Technologies, Palo Alto, CA, USA) by using a RNA 6000 Nano kit (Agilent). The percentage of miRNAs (10–40 nucleotides) was estimated with the same equipment by also analysing 1 µl total RNA in a Small RNA Chip (Agilent). The small RNAs were purified with the PureLink miRNA Isolation Kit (Invitrogen, Carlsbad, CA, USA) and samples were concentrated with a speed vacDNA. 3 µl (10 ng approximately) of miRNA were used for next-generation sequencing. Quality of RNA was assessed by 260 nm absorbance with a Nanodrop ND-1000 spectrophotometer (NanoDrop Technologies Inc, Wilmington, DE, USA), by using Qubit fluorescence (Invitrogen), and by capillary electrophoresis with an Agilent 2100 Bioanalyzer (Agilent Technologies). Fragment libraries compatible with the SOLiD platform were obtained from small RNA by following the recommendations provided by Life Technologies for SOLiDv4 sequencing (http://www.umassmed.edu/uploadedFiles/nemo/STaR-smallRNAs-QRC.PDF). The quality and quantity of the libraries were assessed by Agilent 2100 Bioanalyzer and Qubit.

The equimolar mixture of the libraries was subjected to emulsion PCR process for clonal amplification of the fragments, followed by an enrichment process and chemical modification to generate microspheres and allow loading in the reaction chamber, as indicated by Life Technologies for SOLiD v4 sequencing. The quality and quantity of the microspheres obtained from the pool of libraries have been estimated taking into account the parameters obtained from the workflow analysis. The reactions to obtain sequences of 35 nucleotides +10 nucleotides have been subsequently carried out (Barcode in SOLiD v4). The quality of the data obtained has been estimated using parameters provided by the SETS software (SOLiD Experimental Tracking System). Mass sequencing reactions of miRNAs and the bioinformatic analysis were carried out by Sistemas Genómicos (Spain) [http://www. sistemasgenomicos.com] and the results are shown as a supplementary table (table S1).

Reads were mapped against the latest version of the human genome (version GRch37/hg19) using the BWA software [31]. Known miRNAs were identified and quantified using BedTools [32] and miRBase (v17). Novel miRNAs were discovered by secondary structure predictions with the mirDeep2 package [33].

### Reverse-Transcription and Quantitative Real-time PCR (qRT-PCR) of miRNAs

Total RNA (3 ng) was reverse-transcribed using the TaqMan® MicroRNA Reverse Transcription kit (Applied Biosystems, Foster City, CA, USA) and the miRNA-specific reverse-transcription primers provided with the TaqMan® MicroRNA Assay (Applied Biosystems, Foster City, CA, USA). For the reverse transcription iCycler™ Thermal cycler (Applied Biosystems, Foster City, CA, USA) was used with the following conditions: 16°C for 30 min; 42°C for 30 min and 85°C for 5 min. 1.33 µl of miRNA-specific cDNA from this reaction was amplified with the TaqMan Universal PCR master mix and the respective specific probe provided in the TaqMan® MicroRNA Assay (Applied Biosystems, Foster City, CA, USA). The targeted miRNA assay sequences were as follows: miR-27b 5′-UUCACAGUGGCUAAGUUCUGC-3′, miR-223’- 5′UGUCAGUUUGUCAAAUACCCCA-3′, miR-224 5′-CAAGUCACUAGUGGUUCCGUU-3′, miR-4772-3p 5′-CCUGCAACUUUGCCUGAUCAGA-3′, and miR- 935 5′-CCAGUUACCGCUUCCGCUACCGC-3′. PCR was performed in an iCycler™–MyiQ™ Real-time PCR Detection System (Applied Biosystems, Foster City, CA, USA). Amplification was performed at 95°C for 10 min, followed by 40 cycles of 95°C for 15 s and 60°C for 1 min. miRNA-148a (sequence 5′-UCAGUGCACUACAGAACUUUGU-3′), a miRNA with a fold change close to 1 in mass sequencing, was used as an endogenous control. All mRNA levels were normalized to the values of miRNA-148a and the results expressed as fold changes of threshold cycle (Ct) value relative to controls using the 2^−ΔΔCt^ method [34].

### Statistical Analyses

The Mann-Whitney U test was used to analyse differences between both responder and non-responder groups regarding anthropometric and biochemical variables. For this purpose, SPSS 15.0 software (SPSS, Chicago, IL) was used. SPSS was also used to perform bivariate correlations and calculate Pearson’s r.

Statistical analyses of high-throughput sequencing results were performed with the DESeq software [35] comparing the expression of each miRNA in the responder and non-responder subjects. An adjusted value of probability was achieved by using the Benjamini-Hochberg method for False Discovery Rate (FDR) correction. In the results section, differences in miRNA expression with an adjusted *P*-value<0.05 were interpreted as significant, whereas those with adjusted *P*-value<0.10 were considered as trends towards significance.

Those miRNAs with a significant fold change of 2 or greater (in terms of absolute value) were mapped by chromosome. Moreover, chromosome X and 14 were clustered based on inter-miRNA distance <100000 (http://mirbase.org) [36]. These results are shown in tables S2 and S3.

Prediction of targets for those miRNAs that showed significant differential expression between responders and non-responders was undertaken by means of two widely used algorithms: miRanda and TargetScan. The software MicroCosm Targets Version 5 [36] and the prediction source http://microRNA.org [37] were used for calculating miRanda algorithm, and the software TargetScanHuman 6.0 in the case of TargetScan algorithm [38]. At the time of writing, miR-4772 was not in the MicroCosm Targets Version 5 and TargetScanHuman 6.0 databases.

## Results

### Body Weight after Hypocaloric Diet Treatment

A statistically higher body weight reduction in the responder group (subjects who lost ≥5% body weight) was observed when compared to the non-responders (subjects who lost ≤5% body weight), being the physical activity pattern similar between both groups (table 1).

**Table 1:** Characteristics of the population at baseline and after the 8-week intervention categorized according to the outcome (responders and non-responders to the energy restriction).

	NON RESPONDERS (n = 5)	RESPONDERS (n = 5)	*P*-value
**Age** (years)	42±2	38±2	0.31
**Initial Body Weight** (kg)	90.3±6.2	95.8±3.9	0.55
**Initial Body Mass Index** (BMI)	34.8±2.1	36.4±1.7	0.69
**Initial % Fat** (Impedance)	46.2±2.1	46.9±1.5	1.00
**Waist Circumference** (cm)	105±5	107±4	0.84
**Hip Circumference** (cm)	120±5	125±4	0.58
**Waist/Hip ratio**	0.88±0.04	0.86±0.01	0.69
**Physical activity** (MET h/week)	12.6±7.5	12.1±8.4	0.85
**Fasting Glucose** (mmol/L)	4.94±0.21	4.56±0.27	0.31
**Systolic Blood Pressure** (mm Hg)	122±5	123±4	1.00
**Diastolic Blood Pressure** (mm Hg)	70±4	62±2	0.15
**Weight Loss** (kg)	3.1±0.7	10.8±0.6	**0.008**

Comparisons between groups were made using Mann-Whitney U test. Values represent mean ± SEM.

### miRNA Expression Profile in PBMC Cells

**Figure 1:**
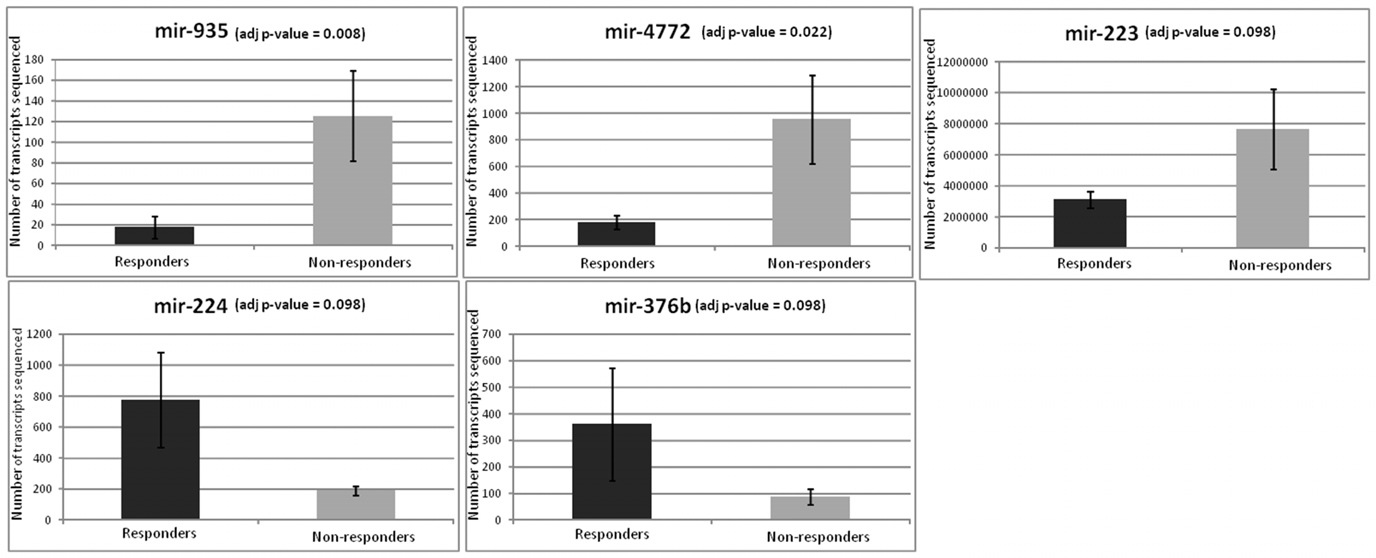
miRNAs differentially expressed between responders and non-responders at baseline (adjusted *P-*value<0.1). The data correspond to the mean of the transcripts sequenced in each group ± SEM.

**Figure 2:**
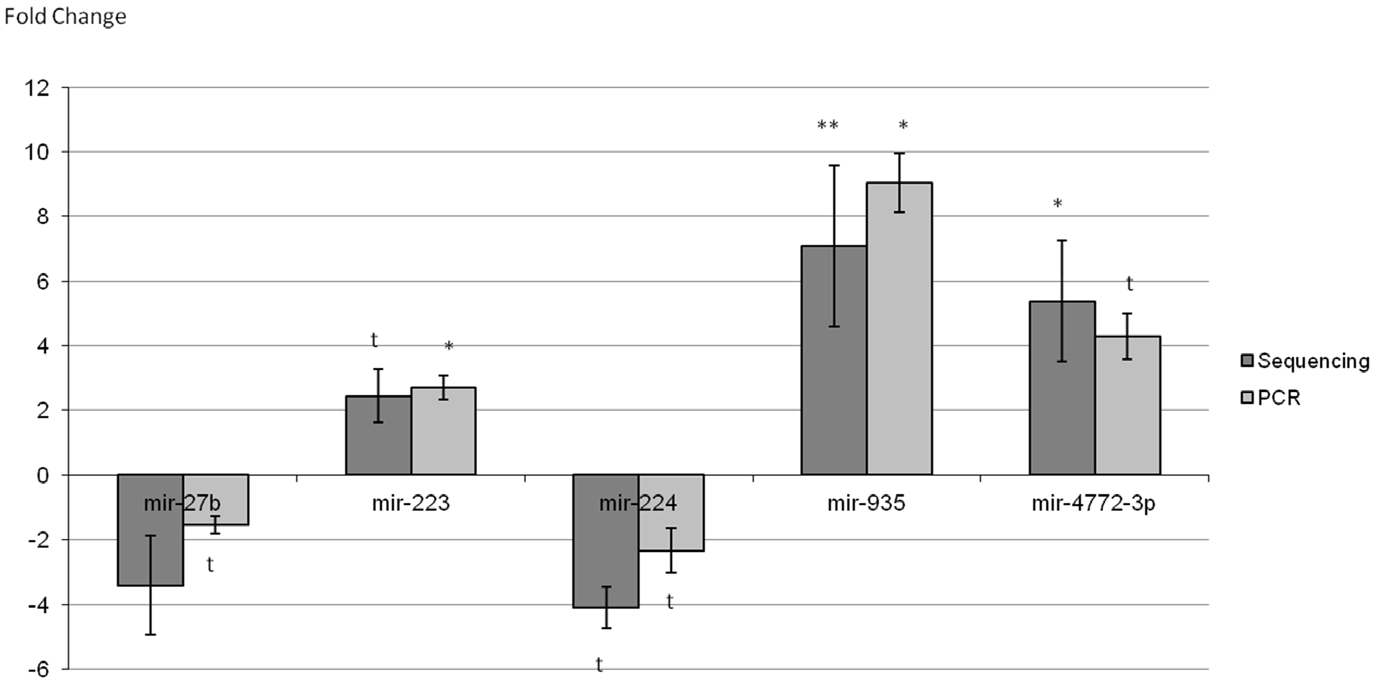
Comparison between the two technologies applied in this work, high-throughput sequencing (dark columns) and RT-PCR (light columns). The expression of mir-27b, mir-223, mir-224, mir-935 and mir-4772-3p is represented as fold change of non-responders with respect to responders at baseline. The data correspond to the mean ± SEM. Comparisons between groups were made using adjusted *P-*value (mass sequencing) and Mann-Whitney U test (RT-PCR). *, p<0.05; t, p<0.1.

Two miRNAs were up-regulated in the non-responder group according to high-throughput sequencing at baseline (figures 1 and 2): mir-935, with a fold change of 7.1 (adjusted *P* -value of 0.008 and a fold change value obtained by qRT-PCR of 9.05, *P* = 0.02), and mir-4772, with a fold change of 5.3 (adjusted *P*-value of 0.022 and a fold change value obtained by qRT-PCR of 4.29, *P* = 0.09). Three other miRNAs were down-regulated but showed an adjusted *P*-value lower than 0.1 and a fold change higher than two or lower than minus two (figures 1 and 2): mir-223, with a fold change of 2.5 (the fold change value obtained by qRT-PCR was 2.72, *P* = 0.03); mir-224, with a fold change of −4.1 (the fold change value obtained by qRT-PCR was −2.33, *P* = 0.08); and mir-376b, with a fold change of −4.2.

Six other transcripts are shown in table 2 because, according to high-throughput sequencing, they presented differential expression between responders and non-responders before using the Benjamini-Hochberg correction for multiple tests (*P*-value<0.005 and fold change of 2.9 or greater in terms of absolute value, but an adjusted *P*-value higher than 0.1). Two of them were overexpressed, being mir-183 the most up-regulated with a fold change of 9.0. Other four miRNAs were down-regulated, showing mir-433 a fold change of −9.6 and mir-27b a fold change of −3.41 (the fold change value obtained by qRT-PCR for mir-27b was −1.54, *P* = 0.09; figure 2).

**Table 2:** miRNA transcripts showing differential miRNA expression between responders and non-responders by using the mass sequencing approach (non-adjusted *P*-value<0.005 and fold change>2.9), but whose adjusted *P*-values were not significant.

miRNAs	Responders	Non-responders	Fold Change	Non-adjusted *P-*value
**mir-183**	4±4	36±13	**8.96**	0.004
**mir-542**	148±24	555±158	**3.75**	0.003
**mir-433**	61±16	6±6	**−9.56**	0.003
**mir-154**	269±105	79±37	**−3.42**	0.005
**mir-27b**	1655±550	485±217	**−3.41**	0.003
**mir-409**	4744±1184	1609±722	**−2.95**	0.005

The data report the mean of the number of transcripts sequenced in each group ± SEM.

Table S4 shows the most expressed miRNAs in PBMC cells based on read counts. The transcript mir-223, which was differentially expressed when comparing responder and non-responder subjects, is the most abundant in this type of cells, followed by mir-150 and mir-126.

### Novel miRNAs

From a total of 102 detected miRNAs with a significant fold change of −2 or greater, fourteen not identified miRNAs (according to miRBase database) were found. Although none of these novel miRNAs showed significant differences between responders and non-responders (table S5), and despite their relatively low expression levels, they deserve a study in the future.

### Prediction of Targets for miRNAs

The predicted gene targets for miRNAs were carried out for miR-935, miR-223, miR-224, miR-376b, miR-542, miR-433, miR-154 miR-27b, miR-409, miR-183, miR-542-3p and miR-4772-3p with miRanda and TargetScan algorithms. With the exception of miR-542, predicted targets related to obesity and its co-morbidities were found in these miRNAs (table 3). In the table S6, the list of target mRNAs of each differentially expressed miRNA has been expanded, including the most relevant obesity-related genes putatively regulated by each miRNA.

**Table 3:** Short selection of target mRNAs of the microRNAs with differences in expression between responders and non-responders to the energy-restricted.

miRNA	Sequence	miRanda algorithm		TargetScan algorithm
	(source mirbase.org )	MicroCosm	microRNA	TargetScan
**hsa-mir-935**	GGCGGGGGCGCGGGCGGCAGUGGCGGGAGCGGCCCCUCGGCCAUCCUCCGUCUGCCCAGUUACCGCUUCCGCUACCGCCGCCGCUCCCGCU	HDAC1, ADRA1A, LPL, RBP5	HIF1A	ADRB1
**hsa-mir-223**	CCUGGCCUCCUGCAGUGCCACGCUCCGUGUAUUUGACAAGCUGAGUUGGACACUCCAUGUGGUAGAGUGUCAGUUUGUCAAAUACCCCAAGUGCGGCACAUGCUUACCAG	IGFL2c, DNMT1, HDAC2, HDAC8, SIRT5	LIPG	IGF1R, HDAC4
**hsa-mir-224**	GGGCUUUCAAGUCACUAGUGGUUCCGUUUAGUAGAUGAUUGUGCAUUGUUUCAAAAUGGUGCCCUAGUGACUACAAAGCCC	MC3R, SREBF1	ACAT1	IRS2
**hsa-mir-376b**	CAGUCCUUCUUUGGUAUUUAAAACGUGGAUAUUCCUUCUAUGUUUACGUGAUUCCUGGUUAAUCAUAGAGGAAAAUCCAUGUUUUCAGUAUCAAAUGCUG	FABP4, CPT2	LIPH, PPARG, G6PC2, HDAC9, NR3C1	HDAC9, IGF1R
**hsa-mir-433**	CCGGGGAGAAGUACGGUGAGCCUGUCAUUAUUCAGAGAGGCUAGAUCCUCUGUGUUGAGAAGGAUCAUGAUGGGCUCCUCGGUGUUCUCCAGG	HDAC6, ADRA1A, IGFBP1, ACOX2, LPL, LIPE	GCLC, FRZB,	ADRA1A, LEPR
**has-mir-154**	GUGGUACUUGAAGAUAGGUUAUCCGUGUUGCCUUCGCUUUAUUUGUGACGAAUCAUACACGGUUGACCUAUUUUUCAGUACCAA	ADRA1A, IGF1		
**hsa-mir-27b**	ACCUCUCUAACAAGGUGCAGAGCUUAGCUGAUUGGUGAACAGUGAUUGGUUUCCGCUUUGUUCACAGUGGCUAAGUUCUGCACCUGAAGAGAAGGUG	PPARG, INSR, MC4R, SIRT5	SCD5, ACAT1,	PPARG, INSR, IRS1, CNR1, PPARGC1B, IGF1
**hsa-mir-409**	UGGUACUCGGGGAGAGGUUACCCGAGCAACUUUGCAUCUGGACGACGAAUGUUGCUCGGUGAACCCCUUUUCGGUAUCA		FZD3, PPARGC1A, APOF, AGPAT9	
**has-mir-183**	CCGCAGAGUGUGACUCCUGUUCUGUGUAUGGCACUGGUAGAAUUCACUGUGAACAGUCUCAGUCAGUGAAUUACCGAAGGGCCAUAAACAGAGCAGAGACAGAUCCACGA	HDAC10, HDAC8, HDAC6, CRTC2, DGKH, GK5, PLB1, CPT2	ACAD8	PEX19
**hsa-mir-542-3p**	UGUGACAGAUUGAUAACUGAAA	IGFBP1, IGFBP6, FABP3, PLA2G4B, LIPA, APOL5, ELOVL3, SLC27A3, PPARD, TNF	FABP3	GDPD4, PLA2R1,
**hsa-mir-4772-3p**	CCUGCAACUUUGCCUGAUCAGA			ACSL1, PDK3, PPARGC1A, ERLIN2, IGF2BP1, IGF2, LRP8, CREBBP

ACAD: acyl-CoA dehydrogenase family; ACAT: acetyl-coa acetyltransferase; ACOX: acyl-coenzyme A oxidase, peroxisomal; ACSL: acyl-CoA synthetase long-chain family member; ADRA1A: alpha-1A adrenergic receptor; ADRB1: beta-1 adrenergic receptor; AGPAT: 1-acylglycerol-3-phosphate o-acyltransferase; APO: apolipoprotein; CREBBP: CREB binding proteína; CNR: cannabinoid receptor; CPT: Carnitine O-palmitoyltransferase, mitochondrial precursor; CRTC: transducer of regulated cAMP response element-binding protein (CREB); DGKH : diacylglycerol kinase eta; DNMT: DNA (cytosine-5)-methyltransferase; ELOVL: elongation of very long chain fatty acids protein; ERLIN: ER lipid raft associated; FABP: fatty acid-binding protein, adipocyte; FRZB: frizzled-related protein; FZD: frizzled, drosophila, homolog of; GCLC: glutamate-cysteine ligase, catalytic subunit; GDPD: glycerophosphodiester phosphodiesterase domain containing; GIPR: gastric inhibitory polypeptide receptor; GK: glycerol kinase; G6PC: glucose-6-phosphatase, catalytic; HDAC: histone deacetylase; HIF1A: hypoxia-inducible factor; IGF1: insulin-like growth factor 1; IGF1R: insulin-like growth factor 1 receptor; IGFBP: insulin-like growth factor-binding protein precursor; IGFL2c: insulin growth factor-like family member 2 precursor; INSR: insulin receptor; IRS: insulin receptor substrate; LEPR: leptin receptor; LIPA: lysosomal acid lipase/cholesteryl ester hydrolase precursor; LIPE: hormone-sensitive lipase; LIPG: factor 1, alpha subunit; lipase, endothelial; LIPH: lipase h; LRP: Low density lipoprotein receptor-related protein, apolipoprotein e receptor;LPL: Lipoprotein lipase precursor; MCR: Melanocortin receptor; NR3C1: glucocorticoid receptor; PDK: pyruvate dehydrogenase kinase, isozyme; PEX: peroxisomal biogenesis factor; PLA2G4B: cytosolic phospholipase A2 beta; PLA2R1: phospholipase A2 receptor 1; PLB: phospholipase B; PPARD: Peroxisome proliferator-activated receptor delta; PPARG: Peroxisome proliferator-activated receptor gamma; PPARGC: peroxisome proliferator-activated receptor gamma, coactivator; RBP: retinol-binding protein, cellular; SLC27A: long-chain fatty acid transport protein; SCD: stearoyl-coa desaturase; SIRT5: NAD-dependent deacetylase sirtuin-5; SREBF1: sterol regulatory element-binding protein 1; TNF: tumor necrosis factor precursor.

### Chromosomal Localization of miRNAs

Sequenced miRNAs with a significant fold change of 2 or greater (in absolute values) have been mapped by chromosome and shown as supplementary data (tables S2 and S3) accompanied by the differences between responders and non-responders. Most of the miRNAs, as determined by the total number of reads mapped per chromosome, are located in the chromosomes 14 and X. When the miRNAs were clustered based on chromosome location and, in the case of chromosomes X and 14, on inter-miRNA distance <100000 (according to miRBase), three clusters located in chromosomes 9, X and 18 were significantly up-regulated. Another three clusters were significantly down-regulated, two of them in chromosome X and one in chromosome 7.

### Associations/correlations between miRNAs and Anthropometric Measurements or Indexes

**Figure 3:**
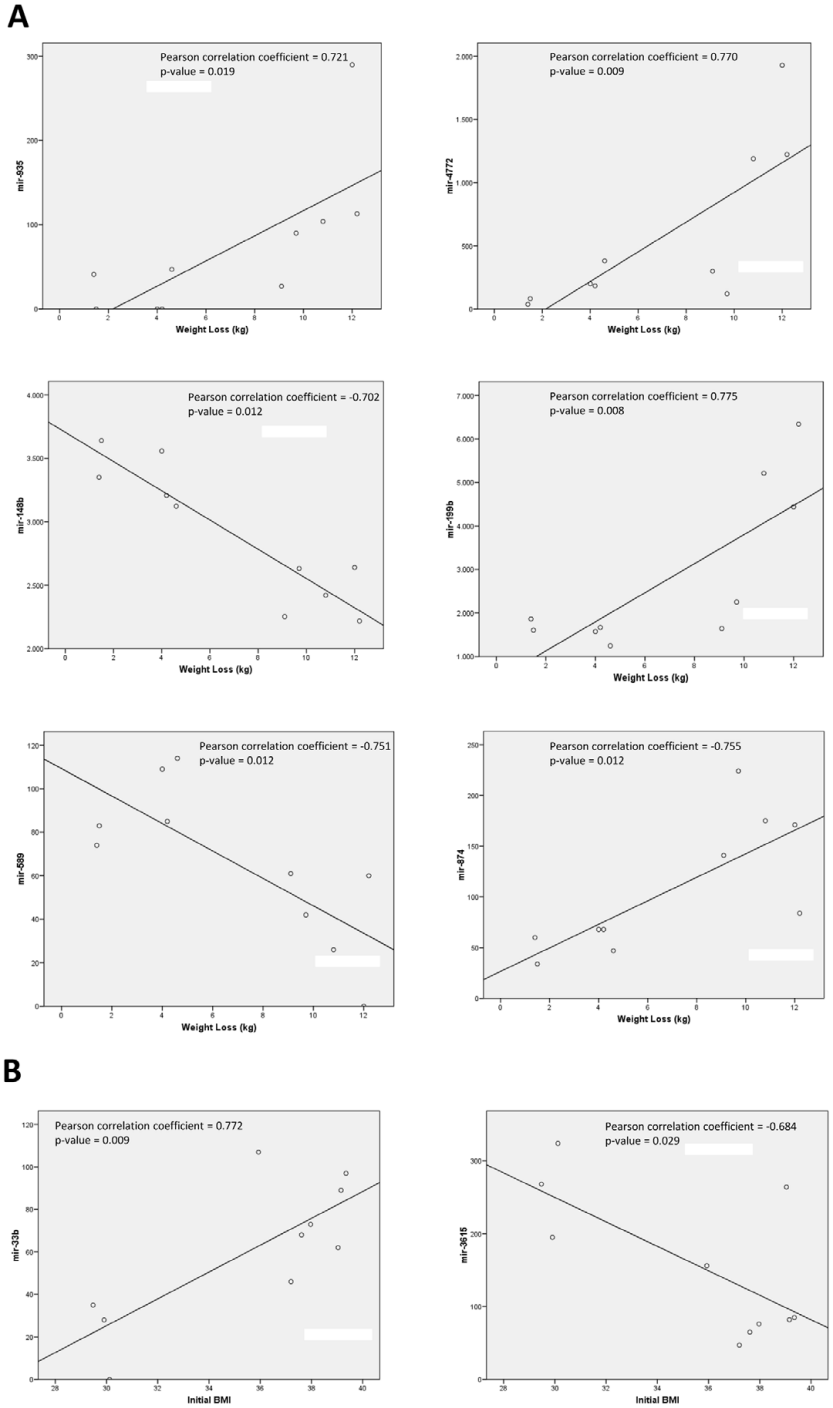
a. Associations between the expression of some miRNAs and body weight change during the energy restriction period. miRNA expression as number of transcripts sequenced. Pearson product-moment correlation coefficient has been applied. **b.** Associations between the expression of some miRNAs and the baseline body mass index (BMI) of the subjects. miRNA expression as number of transcripts sequenced. Pearson product-moment correlation coefficient has been applied.

Both mir-935 and mir-4772 showed close associations with the magnitude of weight loss (figure 3a, with Pearson correlation coefficients of 0.721 and 0.770, respectively). Interestingly, similar results were observed with other miRNAs presenting low *P*-values in the non-adjusted test, such as mir-874 (*P* = 0.014), mir-199b (*P* = 0.020), mir-766 (*P* = 0. 052), mir-589 (*P* = 0.076), and mir-148b (*P* = 0.389). Other miRNAs differentially expressed between responders and non-responders shown in figure 1, as is the case of mir-223 and mir-224, evidenced a trend towards significance in their correlation with the magnitude of weight loss (Pearson correlation coefficients of 0.597 and 0.599 and *P*-values of 0.069 and 0.067, respectively).

There were no baseline differences between the participants in the study regarding their anthropometric and biochemical measurements (table 1). However, we wanted to know if the expression of some miRNAs in PBMCs were associated with initial BMI, the most used parameter of adiposity (figure 3b). Moderate associations were found between the expression of some miRNAs and this phenotypic marker of adiposity, with mir-3615 and mir-33b being the more relevant. These miRNAs associated to initial body weight could be candidates for being implicated in obesity onset and complications, although it should be elucidated in later studies.

**Figure 4:**
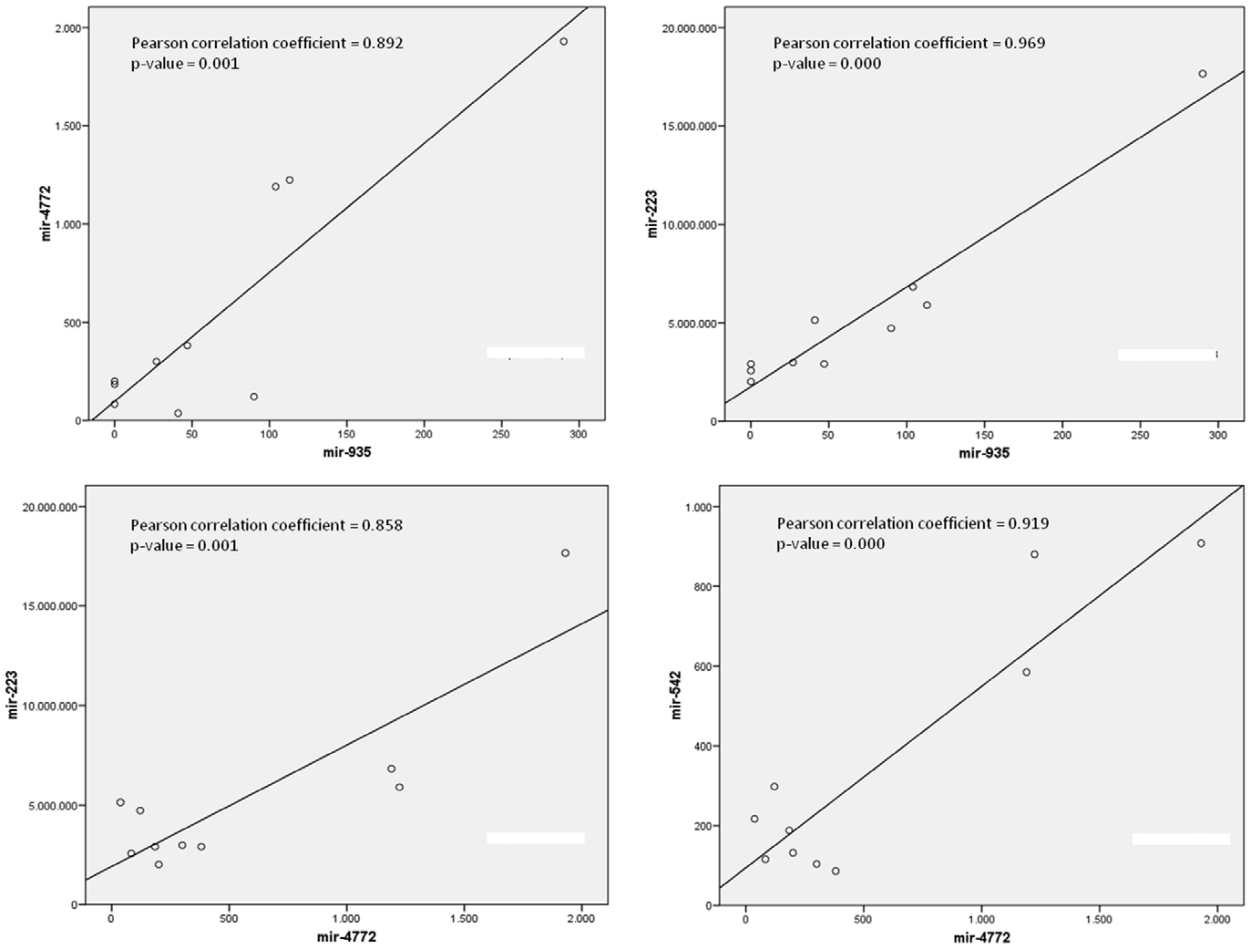
Associations between the expression of different miRNAs that are involved in the response to the dietary treatment. Data represent the number of transcripts sequenced. Pearson product-moment correlation coefficient has been applied.

It is noteworthy that the expression of some of the analysed miRNAs was highly correlated among them, as observed when comparing the miRNAs that were more associated with weight loss, such as mir-935, mir-4772, mir-223, mir-409 and mir-27b (figure 4).

## Discussion

The current research is pioneer in the use of high-throughput sequencing in the search for expression biomarkers in obesity, by determining the whole miRNA transcriptome in human PBMCs. The current article employed a similar approach that Ahn *et al*. [39] when determined the miRNA transcriptome in the newborn mouse ovaries. In our case, we studied the miRNA transcriptome in peripheral blood cells of ten obese women that followed a weight-loss program, with 5 of them categorized as high responders and the other 5 as non-responders, according to Campion et al. [40]. This kind of cells has been selected because they are widely and successfully used in the search for non-invasive biomarkers as surrogates of tissues that are not easily accessible, such as adipose tissue [41]. We found 5 miRNAs whose expression was different at baseline between both groups (with an adjusted *p*-value lower than 0.1). Among them, only mir-223 (fold change of 2.5) and mir-224 (fold change of −4.1) had been previously related to obesity. Thus, mir-223 has been reported to regulate the expression of many genes, as for example GLUT4 [42], and is overexpressed in epididymal adipocytes of obese mice [43] and down-regulated in plasma of diabetic patients [40]. On the other hand, mir-224 is the only differentially expressed miRNA that has been previously related to adiposity, being up-regulated in 3T3-L1 mature adipocytes 9 days post induction when compared with preadipocytes [44].

Regarding the expression of the other 3 miRNAs shown in figure 1 (mir-935, mir-4772 and mir-376b), this study is apparently the first that finds differences in PBMC cells and that relates their expression with the susceptibility to lose weight. More studies (*in vitro* and in animals) are necessary to confirm a role of these 3 transcripts in obesity and energy metabolism.

Some of the miRNAs reported in table 2 have been previously associated with different diseases, especially cancer. Among them, there are three miRNAs (mir-183, mir-433 and mir-27b) that seem to play an important role in the regulation of gene expression in metabolic disorders. Thus, regarding mir-183 (fold change of 9.0), the incubation of 3T3-L1 adipocytes with insulin inhibits its expression [45]. mir-433 (fold change of −9,6) is activated by the estrogen receptor ESRRG, in which several SNPs have been associated with higher risk of type 2 diabetes by GWAS [46] and that acts as a co-activator of the demethylating enzyme Dnmt1 [47]. Although most articles studying this specific miRNA are focused on cancer [48], it is also differentially expressed in liver of patients with non-alcoholic fatty liver disease [49]. Finally, mir-27b (fold change of −3,4), which is an oxidative stress-responsive miRNA that suppresses lipopolysaccharide-induced activation of NFκB [50], is up-regulated in epididymal adipocytes of obese mice [43] and impairs human adipocyte differentiation by targeting PPARγ [51].

The moderate correlations observed between the expression of the different miRNAs in PBMCs and the BMI at baseline (figure 3b) suggest that small differences in adiposity do not appears to strongly affect miRNA expression in this kind of cells. However, the magnitude of weight loss strongly correlated with the expression of several miRNAs at baseline (figure 3a), suggesting a role of these miRNAs in body weight regulation, energy homeostasis and response to the diet. The significant correlations found between the expression of different miRNAs (figure 4) suggest that, at least in PBMCs, their expression could be regulated by common factors and even that they might share similar regulatory functions.

Some of the potential targets of the miRNAs identified in the present study are represented in table 3. Those that have been extensively related to obesity, energy homeostasis and insulin signalling, as well as those that can act at an epigenetic level, have been reflected in the table. This is the case of genes like peroxisome proliferator-activated receptor gamma (PPARG), glucocorticoid receptor (NR3C1), hypoxia-inducible factor 1, alpha subunit (HIF1A), gastric inhibitory polypeptide receptor (GIPR), histone deacetylases (HDAC) and methyltransferases (DNMT). None of the miRNAs shown in table 3 have been previously studied in relation to the expression of these genes in models of obesity or weight regulation. So that, *in vitro* studies with siRNAs or addition of miRNAs to the culture medium of 3T3-L1 cells, primary adipocytes, monocytes, macrophages or other key cell types in the development of obesity, are needed to elucidate the real importance of these miRNAs on the regulation of the expression of these genes. However, previous studies have revealed an interaction between some miRNAs, none of them differentially expressed in our study, and the expression of HIF1A (miR-210 [52] and miR-101 [53], among many others), PPARG (such as miR-130 [54]), or NR3C1 (including miR-18 and 124a, [55]), suggesting that miRNAs may play an important role in the regulation of these genes and the related pathways. It is noteworthy that some of the analyzed miRNAs have histone deacetylases and methyltransferases as predicted targets, suggesting an epigenetic loop between miRNAs and histone modifications in gene expression regulation.

Some of the highly abundant miRNA transcripts observed in the present study coincide with those reported by Vaz et al. [10] in a previous analysis of microRNA transcriptome in PBMCs by deep sequencing, as for example mir-29a, mir-21, mir-191 or let-7g. However, the disparities could be partially attributed to the difference in technology (SOLiD v4 *versus* SOLEXA).

This is the first work that determines the whole miRNA transcriptome by using high-throughput sequencing in the search for biomarkers related to obesity and body weight regulation. The results of this study stress the importance of miRNA expression in blood cells as biomarkers of weight loss and response to the diet (prognostic biomarkers), which could be used in the design of personalized nutrition. Also, they suggest that the expression of different miRNAs in blood cells might be implicated in the regulation of energy homeostasis and body weight. However, it should be emphasized that some of the miRNA expression differences observed between responders and non-responders could be due to (or influenced by) the diet at baseline or other factors, such as physical activity, genetic background, dietary habits or immune function, that should be analyzed in further studies.

To conclude, the present study proposes the basal expression levels of different miRNAs in blood cells, particularly mir-935, mir-4772, mir-223, mir-224 and mir-376b, as prognostic biomarkers of response to the diet. This research represents a milestone in the design of personalized nutrition by taking into account miRNA expression in blood cells, and opens the door to the study of different miRNAs as novel biomarkers of weight loss.

## Supporting Information

Table S1 **All the miRNA transcripts sequenced and the differences between responders and non-responders (as fold change), categorized by p-value adjusted for multiple comparisons.**
(DOC)

Table S2 **miRNA clusters (according to miRBase) upregulated in peripheral blood cells of non-responders to the low-calorie diet when compared to the responders, categorized by chromosomal location.**
(DOC)

Table S3 **miRNA clusters (according to miRBase) downregulated in peripheral blood cells of non-responders to the low-calorie diet when compared to the responders, categorized by chromosomal location.**
(DOC)

Table S4 **The most expressed miRNA transcripts in PBMC cells, based on read counts, and the chromosome where they are located.**
(DOC)

Table S5 **List of predicted novel miRNAs (according to miRBase) found in peripheral blood mononuclear cells.**
(DOC)

Table S6 **Target mRNAs of the microRNAs with differences in expression between responders and non-responders to the energy-restricted.**
(DOC)
