# Supplementary material for: High-Throughput Sequencing of microRNAs in Peripheral Blood Mononuclear Cells: Identification of Potential Weight Loss Biomarkers
Source: PLoS One. 2013 Jan 15;8(1):e54319. doi: 10.1371/journal.pone.0054319 (PMC3545952; doi:10.1371/journal.pone.0054319)
Supplement: Table S1 — All the miRNA transcripts sequenced and the differences between responders and non-responders (as fold change), categorized by p-value adjusted for multiple comparisons. (DOC) [file pone.0054319.s001.doc]

**Supplementary table 1.** All the miRNA transcripts sequenced and the differences between responders and non-responders (as fold change), categorized by p-value adjusted for multiple comparisons. Analysis of comparisons between groups was undertaken by using DESeq software. An adjusted value of probability was achieved by using Benjamini-Hochberg FDR correction for multiple testing.

| **miRNA** | **Responders**  **(Mean ± SEM)** | | | **Non-Responders**  **(Mean ± SEM)** | | | **Fold Change** | **p-value** | **p-adjusted value** |
| --- | --- | --- | --- | --- | --- | --- | --- | --- | --- |
| hsa-mir-935 | 18 | ± | 11 | 125 | ± | 44 | 7.09 | 0.000 | 0.009 |
| hsa-mir-4772 | 177 | ± | 59 | 953 | ± | 332 | 5.38 | 0.000 | 0.022 |
| hsa-mir-223 | 3108550 | ± | 533782 | 7618398 | ± | 2590465 | 2.45 | 0.001 | 0.098 |
| hsa-mir-224 | 776 | ± | 309 | 189 | ± | 30 | -4.10 | 0.001 | 0.098 |
| hsa-mir-376b | 359 | ± | 213 | 85 | ± | 29 | -4.22 | 0.001 | 0.098 |
| hsa-mir-183 | 4 | ± | 4 | 36 | ± | 13 | 8.96 | 0.004 | 0.141 |
| hsa-mir-542 | 148 | ± | 24 | 555 | ± | 158 | 3.75 | 0.002 | 0.141 |
| hsa-mir-3647 | 0 | ± | 0 | 17 | ± | 11 | 0.00 | 0.005 | 0.141 |
| chr5_108084315_108084426_- | 3 | ± | 3 | 3 | ± | 3 | -1.00 | 0.005 | 0.141 |
| hsa-mir-409 | 4744 | ± | 1184 | 1609 | ± | 722 | -2.95 | 0.005 | 0.141 |
| hsa-mir-136 | 371 | ± | 133 | 110 | ± | 57 | -3.36 | 0.005 | 0.141 |
| hsa-mir-27b | 1655 | ± | 550 | 485 | ± | 217 | -3.41 | 0.003 | 0.141 |
| hsa-mir-154 | 269 | ± | 105 | 79 | ± | 37 | -3.42 | 0.005 | 0.141 |
| hsa-mir-433 | 61 | ± | 16 | 6 | ± | 6 | -9.56 | 0.003 | 0.141 |
| hsa-mir-487b | 2869 | ± | 954 | 971 | ± | 507 | -2.95 | 0.006 | 0.143 |
| hsa-mir-543 | 447 | ± | 206 | 137 | ± | 44 | -3.26 | 0.006 | 0.143 |
| hsa-mir-370 | 161 | ± | 88 | 46 | ± | 38 | -3.50 | 0.007 | 0.144 |
| hsa-mir-153-2 | 5 | ± | 5 | 5 | ± | 5 | 1.00 | 0.009 | 0.154 |
| hsa-mir-376c | 8908 | ± | 4138 | 3446 | ± | 1299 | -2.58 | 0.008 | 0.154 |
| hsa-mir-494 | 2244 | ± | 664 | 776 | ± | 398 | -2.89 | 0.008 | 0.154 |
| hsa-mir-654 | 992 | ± | 474 | 330 | ± | 229 | -3.01 | 0.009 | 0.154 |
| hsa-mir-496 | 114 | ± | 25 | 30 | ± | 16 | -3.78 | 0.009 | 0.154 |
| hsa-mir-874 | 55 | ± | 7 | 159 | ± | 23 | 2.87 | 0.015 | 0.209 |
| hsa-mir-532 | 1605 | ± | 530 | 4145 | ± | 622 | 2.58 | 0.016 | 0.209 |
| hsa-mir-342 | 135235 | ± | 29150 | 281525 | ± | 60554 | 2.08 | 0.014 | 0.209 |
| hsa-mir-337 | 2104 | ± | 649 | 781 | ± | 397 | -2.69 | 0.014 | 0.209 |
| hsa-mir-299 | 243 | ± | 73 | 86 | ± | 48 | -2.83 | 0.017 | 0.209 |
| hsa-mir-485 | 254 | ± | 66 | 89 | ± | 34 | -2.86 | 0.015 | 0.209 |
| hsa-mir-376a-1 | 81 | ± | 25 | 20 | ± | 14 | -4.01 | 0.016 | 0.209 |
| hsa-mir-199b | 1591 | ± | 100 | 3977 | ± | 888 | 2.50 | 0.020 | 0.223 |
| hsa-mir-4742 | 4 | ± | 4 | 3 | ± | 3 | -1.19 | 0.020 | 0.223 |
| hsa-mir-379 | 647 | ± | 235 | 237 | ± | 95 | -2.73 | 0.019 | 0.223 |
| hsa-mir-411 | 64 | ± | 20 | 13 | ± | 13 | -4.75 | 0.019 | 0.223 |
| hsa-mir-539 | 96 | ± | 34 | 29 | ± | 20 | -3.28 | 0.024 | 0.255 |
| hsa-mir-96 | 31 | ± | 11 | 90 | ± | 35 | 2.93 | 0.026 | 0.265 |
| hsa-mir-410 | 85 | ± | 23 | 25 | ± | 16 | -3.41 | 0.026 | 0.265 |
| hsa-mir-582 | 263 | ± | 50 | 683 | ± | 296 | 2.60 | 0.027 | 0.265 |
| hsa-mir-487a | 131 | ± | 44 | 46 | ± | 29 | -2.81 | 0.028 | 0.267 |
| hsa-mir-371b | 5 | ± | 5 | 27 | ± | 11 | 5.95 | 0.033 | 0.294 |
| hsa-mir-424 | 5471 | ± | 519 | 11750 | ± | 3938 | 2.15 | 0.032 | 0.294 |
| hsa-mir-4446 | 4 | ± | 4 | 5 | ± | 5 | 1.14 | 0.034 | 0.294 |
| chr6_31670998_31671109_- | 4 | ± | 4 | 3 | ± | 3 | -1.33 | 0.034 | 0.294 |
| hsa-mir-3614 | 11 | ± | 7 | 42 | ± | 16 | 3.82 | 0.041 | 0.335 |
| hsa-mir-338 | 3063 | ± | 566 | 6597 | ± | 1979 | 2.15 | 0.040 | 0.335 |
| hsa-mir-323 | 415 | ± | 115 | 175 | ± | 77 | -2.37 | 0.043 | 0.335 |
| hsa-mir-382 | 308 | ± | 120 | 129 | ± | 63 | -2.39 | 0.042 | 0.335 |
| hsa-mir-127 | 100 | ± | 41 | 36 | ± | 22 | -2.78 | 0.043 | 0.335 |
| hsa-mir-376a-2 | 39 | ± | 14 | 6 | ± | 6 | -6.47 | 0.045 | 0.340 |
| hsa-mir-766 | 54 | ± | 4 | 14 | ± | 9 | -3.80 | 0.053 | 0.392 |
| hsa-mir-29b-1 | 84 | ± | 26 | 190 | ± | 47 | 2.26 | 0.054 | 0.393 |
| hsa-mir-503 | 56 | ± | 18 | 15 | ± | 10 | -3.61 | 0.056 | 0.405 |
| hsa-let-7e | 452 | ± | 42 | 202 | ± | 79 | -2.23 | 0.060 | 0.419 |
| hsa-mir-3065 | 3 | ± | 3 | 5 | ± | 5 | 1.41 | 0.061 | 0.424 |
| hsa-mir-3607 | 64 | ± | 12 | 141 | ± | 46 | 2.22 | 0.065 | 0.438 |
| hsa-mir-18a | 2452 | ± | 956 | 1187 | ± | 182 | -2.07 | 0.066 | 0.441 |
| hsa-mir-369 | 263 | ± | 67 | 121 | ± | 60 | -2.17 | 0.069 | 0.449 |
| hsa-mir-493 | 74 | ± | 21 | 27 | ± | 21 | -2.77 | 0.070 | 0.451 |
| hsa-mir-2115 | 6 | ± | 6 | 25 | ± | 13 | 4.50 | 0.078 | 0.477 |
| hsa-mir-589 | 93 | ± | 8 | 38 | ± | 11 | -2.46 | 0.076 | 0.477 |
| hsa-mir-1185-1 | 72 | ± | 29 | 26 | ± | 21 | -2.71 | 0.077 | 0.477 |
| hsa-mir-101-1 | 90 | ± | 35 | 186 | ± | 50 | 2.08 | 0.082 | 0.480 |
| hsa-mir-99b | 1456 | ± | 710 | 721 | ± | 109 | -2.02 | 0.084 | 0.480 |
| hsa-mir-551b | 155 | ± | 41 | 72 | ± | 40 | -2.16 | 0.081 | 0.480 |
| hsa-mir-3940 | 94 | ± | 36 | 40 | ± | 13 | -2.38 | 0.084 | 0.480 |
| hsa-mir-141 | 6 | ± | 6 | 5 | ± | 5 | -1.33 | 0.085 | 0.481 |
| hsa-mir-204 | 110 | ± | 43 | 223 | ± | 46 | 2.04 | 0.090 | 0.486 |
| hsa-mir-29a | 22945 | ± | 5398 | 39474 | ± | 6150 | 1.72 | 0.094 | 0.486 |
| chr21_43951195_43951304_+ | 4 | ± | 4 | 6 | ± | 6 | 1.50 | 0.088 | 0.486 |
| hsa-mir-130a | 18039 | ± | 5909 | 10251 | ± | 4690 | -1.76 | 0.094 | 0.486 |
| chr9_132631860_132631968_+ | 5 | ± | 5 | 3 | ± | 3 | -1.86 | 0.092 | 0.486 |
| hsa-mir-152 | 1469 | ± | 183 | 743 | ± | 83 | -1.98 | 0.093 | 0.486 |
| hsa-mir-150 | 352929 | ± | 104340 | 575778 | ± | 93689 | 1.63 | 0.096 | 0.487 |
| chr9_135821064_135821189_+ | 9 | ± | 5 | 30 | ± | 13 | 3.51 | 0.100 | 0.494 |
| hsa-mir-1294 | 5 | ± | 5 | 7 | ± | 7 | 1.37 | 0.104 | 0.494 |
| chr16_81567491_81567601_+ | 7 | ± | 4 | 5 | ± | 5 | -1.33 | 0.103 | 0.494 |
| chr7_148638560_148638674_+ | 19915 | ± | 5846 | 11505 | ± | 3723 | -1.73 | 0.101 | 0.494 |
| hsa-mir-19b-2 | 28 | ± | 8 | 4 | ± | 4 | -7.31 | 0.101 | 0.494 |
| hsa-mir-1229 | 67 | ± | 16 | 27 | ± | 13 | -2.51 | 0.106 | 0.495 |
| hsa-mir-192 | 4291 | ± | 918 | 7679 | ± | 1166 | 1.79 | 0.110 | 0.509 |
| hsa-mir-195 | 341 | ± | 44 | 174 | ± | 46 | -1.97 | 0.111 | 0.509 |
| hsa-mir-452 | 105 | ± | 34 | 50 | ± | 13 | -2.12 | 0.117 | 0.521 |
| hsa-mir-190 | 45 | ± | 7 | 15 | ± | 15 | -3.10 | 0.117 | 0.521 |
| hsa-mir-3174 | 4 | ± | 4 | 16 | ± | 10 | 3.95 | 0.122 | 0.524 |
| chr2_232321140_232321252_- | 236 | ± | 47 | 451 | ± | 84 | 1.91 | 0.127 | 0.524 |
| chr19_49994413_49994519_+ | 136 | ± | 30 | 256 | ± | 111 | 1.88 | 0.130 | 0.524 |
| hsa-mir-126 | 440972 | ± | 82626 | 278154 | ± | 77547 | -1.59 | 0.129 | 0.524 |
| hsa-mir-26b | 6391 | ± | 2351 | 3652 | ± | 1058 | -1.75 | 0.124 | 0.524 |
| hsa-mir-584 | 2570 | ± | 809 | 1403 | ± | 478 | -1.83 | 0.122 | 0.524 |
| hsa-mir-135b | 6 | ± | 6 | 3 | ± | 3 | -2.00 | 0.126 | 0.524 |
| chr1_226793200_226793309_+ | 6 | ± | 6 | 3 | ± | 3 | -2.23 | 0.129 | 0.524 |
| hsa-mir-656 | 27 | ± | 9 | 5 | ± | 5 | -5.95 | 0.128 | 0.524 |
| hsa-mir-191 | 106251 | ± | 17672 | 167447 | ± | 15838 | 1.58 | 0.133 | 0.529 |
| hsa-mir-450b | 26 | ± | 7 | 57 | ± | 19 | 2.22 | 0.142 | 0.557 |
| chr14_60113648_60113758_- | 6 | ± | 6 | 3 | ± | 3 | -2.38 | 0.151 | 0.588 |
| chr2_64567873_64567982_- | 2041 | ± | 642 | 1158 | ± | 523 | -1.76 | 0.153 | 0.591 |
| chr22_29729190_29729303_- | 452 | ± | 119 | 817 | ± | 185 | 1.81 | 0.159 | 0.607 |
| hsa-mir-3653 | 453 | ± | 120 | 818 | ± | 185 | 1.80 | 0.161 | 0.608 |
| hsa-mir-138-1 | 5 | ± | 5 | 14 | ± | 9 | 2.69 | 0.195 | 0.612 |
| chr9_35710643_35710753_- | 61 | ± | 26 | 111 | ± | 29 | 1.82 | 0.179 | 0.612 |
| hsa-mir-34a | 101 | ± | 28 | 176 | ± | 15 | 1.74 | 0.189 | 0.612 |
| hsa-mir-32 | 1184 | ± | 380 | 2047 | ± | 350 | 1.73 | 0.173 | 0.612 |
| hsa-mir-556 | 3 | ± | 3 | 0 | ± | 0 | 0.00 | 0.202 | 0.612 |
| hsa-mir-548e | 3 | ± | 3 | 0 | ± | 0 | 0.00 | 0.202 | 0.612 |
| hsa-mir-412 | 3 | ± | 3 | 0 | ± | 0 | 0.00 | 0.201 | 0.612 |
| hsa-mir-200b | 3 | ± | 3 | 0 | ± | 0 | 0.00 | 0.201 | 0.612 |
| hsa-mir-147b | 3 | ± | 3 | 0 | ± | 0 | 0.00 | 0.201 | 0.612 |
| hsa-mir-1255a | 3 | ± | 3 | 0 | ± | 0 | 0.00 | 0.180 | 0.612 |
| hsa-mir-1197 | 3 | ± | 3 | 0 | ± | 0 | 0.00 | 0.201 | 0.612 |
| chr2_163918011_163918120_- | 3 | ± | 3 | 0 | ± | 0 | 0.00 | 0.180 | 0.612 |
| chr1_150207047_150207157_+ | 3 | ± | 3 | 0 | ± | 0 | 0.00 | 0.180 | 0.612 |
| hsa-mir-23b | 26489 | ± | 5355 | 17192 | ± | 2612 | -1.54 | 0.188 | 0.612 |
| hsa-mir-221 | 8118 | ± | 3196 | 5064 | ± | 1589 | -1.60 | 0.184 | 0.612 |
| hsa-mir-323b | 1730 | ± | 523 | 1037 | ± | 392 | -1.67 | 0.202 | 0.612 |
| chr7_142157319_142157427_- | 816 | ± | 310 | 465 | ± | 148 | -1.75 | 0.179 | 0.612 |
| hsa-mir-181d | 163 | ± | 46 | 93 | ± | 15 | -1.76 | 0.192 | 0.612 |
| hsa-mir-331 | 270 | ± | 61 | 150 | ± | 18 | -1.80 | 0.165 | 0.612 |
| chr17_75085482_75085599_+ | 8 | ± | 5 | 4 | ± | 4 | -1.81 | 0.180 | 0.612 |
| hsa-let-7f-1 | 124 | ± | 32 | 67 | ± | 21 | -1.84 | 0.177 | 0.612 |
| chr3_49058038_49058157_+ | 90 | ± | 14 | 46 | ± | 18 | -1.94 | 0.180 | 0.612 |
| hsa-mir-134 | 47 | ± | 20 | 20 | ± | 13 | -2.37 | 0.195 | 0.612 |
| chr2_16612710_16612819_- | 27 | ± | 13 | 7 | ± | 7 | -3.91 | 0.202 | 0.612 |
| chr1_55784327_55784436_+ | 22 | ± | 11 | 3 | ± | 3 | -6.35 | 0.206 | 0.617 |
| hsa-mir-21 | 276145 | ± | 63069 | 188955 | ± | 85279 | -1.46 | 0.211 | 0.621 |
| hsa-mir-30a | 257 | ± | 57 | 152 | ± | 12 | -1.69 | 0.213 | 0.621 |
| hsa-mir-146a | 711 | ± | 351 | 420 | ± | 60 | -1.69 | 0.210 | 0.621 |
| hsa-mir-758 | 37 | ± | 16 | 14 | ± | 9 | -2.63 | 0.214 | 0.621 |
| hsa-mir-4632 | 4 | ± | 4 | 0 | ± | 0 | 0.00 | 0.223 | 0.629 |
| hsa-mir-3676 | 4 | ± | 4 | 0 | ± | 0 | 0.00 | 0.223 | 0.629 |
| hsa-mir-107 | 18239 | ± | 3829 | 12113 | ± | 1293 | -1.51 | 0.222 | 0.629 |
| hsa-mir-1249 | 75 | ± | 23 | 40 | ± | 11 | -1.88 | 0.223 | 0.629 |
| hsa-mir-193a | 61 | ± | 24 | 105 | ± | 51 | 1.71 | 0.229 | 0.631 |
| hsa-mir-4676 | 9 | ± | 6 | 5 | ± | 5 | -1.76 | 0.229 | 0.631 |
| hsa-mir-668 | 21 | ± | 6 | 4 | ± | 4 | -5.89 | 0.227 | 0.631 |
| hsa-mir-197 | 44 | ± | 6 | 77 | ± | 23 | 1.76 | 0.241 | 0.655 |
| hsa-mir-10a | 433 | ± | 105 | 263 | ± | 102 | -1.64 | 0.240 | 0.655 |
| hsa-mir-664 | 3257 | ± | 427 | 2090 | ± | 329 | -1.56 | 0.247 | 0.664 |
| hsa-mir-652 | 3230 | ± | 417 | 2079 | ± | 848 | -1.55 | 0.250 | 0.665 |
| hsa-mir-381 | 8 | ± | 5 | 3 | ± | 3 | -2.79 | 0.251 | 0.665 |
| hsa-mir-1287 | 55 | ± | 7 | 28 | ± | 18 | -1.94 | 0.262 | 0.690 |
| hsa-mir-942 | 4 | ± | 4 | 0 | ± | 0 | 0.00 | 0.268 | 0.698 |
| hsa-mir-219-1 | 4 | ± | 4 | 0 | ± | 0 | 0.00 | 0.269 | 0.698 |
| chr11_64507262_64507372_- | 36 | ± | 4 | 15 | ± | 10 | -2.31 | 0.275 | 0.704 |
| chr4_147329729_147329840_+ | 19 | ± | 9 | 3 | ± | 3 | -6.78 | 0.274 | 0.704 |
| hsa-mir-618 | 10 | ± | 6 | 24 | ± | 11 | 2.43 | 0.295 | 0.708 |
| chr20_37078060_37078169_+ | 19 | ± | 6 | 38 | ± | 15 | 1.98 | 0.292 | 0.708 |
| chr10_43966653_43966777_+ | 7 | ± | 7 | 12 | ± | 12 | 1.76 | 0.298 | 0.708 |
| hsa-mir-132 | 125 | ± | 18 | 193 | ± | 18 | 1.55 | 0.297 | 0.708 |
| hsa-mir-4521 | 4 | ± | 4 | 0 | ± | 0 | 0.00 | 0.292 | 0.708 |
| hsa-mir-380 | 4 | ± | 4 | 0 | ± | 0 | 0.00 | 0.291 | 0.708 |
| chr14_95623985_95624094_+ | 4 | ± | 4 | 0 | ± | 0 | 0.00 | 0.292 | 0.708 |
| chr11_8705755_8705865_+ | 4 | ± | 4 | 0 | ± | 0 | 0.00 | 0.292 | 0.708 |
| hsa-mir-423 | 1501 | ± | 262 | 985 | ± | 243 | -1.52 | 0.295 | 0.708 |
| hsa-mir-432 | 40 | ± | 13 | 19 | ± | 19 | -2.12 | 0.283 | 0.708 |
| chr10_120819507_120819616_- | 9 | ± | 6 | 3 | ± | 3 | -2.59 | 0.292 | 0.708 |
| hsa-mir-876 | 7 | ± | 7 | 11 | ± | 7 | 1.66 | 0.314 | 0.716 |
| hsa-mir-143 | 1106 | ± | 350 | 1665 | ± | 330 | 1.51 | 0.311 | 0.716 |
| hsa-mir-140 | 37664 | ± | 5494 | 51407 | ± | 9207 | 1.36 | 0.326 | 0.716 |
| hsa-mir-1343 | 4 | ± | 4 | 0 | ± | 0 | 0.00 | 0.316 | 0.716 |
| chr2_25109063_25109172_- | 4 | ± | 4 | 0 | ± | 0 | 0.00 | 0.316 | 0.716 |
| hsa-mir-222 | 6867 | ± | 1838 | 4836 | ± | 1005 | -1.42 | 0.328 | 0.716 |
| hsa-mir-186 | 885 | ± | 171 | 588 | ± | 72 | -1.50 | 0.324 | 0.716 |
| hsa-mir-335 | 271 | ± | 44 | 179 | ± | 43 | -1.51 | 0.329 | 0.716 |
| hsa-mir-2355 | 666 | ± | 84 | 441 | ± | 73 | -1.51 | 0.327 | 0.716 |
| hsa-mir-454 | 389 | ± | 78 | 257 | ± | 48 | -1.51 | 0.326 | 0.716 |
| hsa-mir-421 | 272 | ± | 67 | 176 | ± | 46 | -1.54 | 0.305 | 0.716 |
| hsa-mir-570 | 30 | ± | 12 | 13 | ± | 6 | -2.40 | 0.308 | 0.716 |
| chr19_50295100_50295209_+ | 23 | ± | 12 | 7 | ± | 7 | -3.14 | 0.312 | 0.716 |
| hsa-mir-95 | 9 | ± | 6 | 3 | ± | 3 | -3.38 | 0.328 | 0.716 |
| hsa-mir-933 | 5 | ± | 5 | 0 | ± | 0 | 0.00 | 0.339 | 0.720 |
| hsa-mir-4787 | 5 | ± | 5 | 0 | ± | 0 | 0.00 | 0.340 | 0.720 |
| hsa-mir-34b | 5 | ± | 5 | 0 | ± | 0 | 0.00 | 0.339 | 0.720 |
| hsa-mir-502 | 30 | ± | 13 | 14 | ± | 14 | -2.22 | 0.339 | 0.720 |
| hsa-mir-4662a | 16 | ± | 12 | 3 | ± | 3 | -4.83 | 0.341 | 0.720 |
| hsa-mir-193b | 101 | ± | 16 | 150 | ± | 33 | 1.49 | 0.345 | 0.721 |
| chr9_130502375_130502487_- | 21 | ± | 15 | 6 | ± | 6 | -3.25 | 0.344 | 0.721 |
| chr18_3885333_3885442_+ | 7 | ± | 7 | 11 | ± | 11 | 1.56 | 0.349 | 0.721 |
| hsa-mir-28 | 273 | ± | 117 | 405 | ± | 96 | 1.48 | 0.352 | 0.721 |
| hsa-mir-374a | 387 | ± | 24 | 568 | ± | 147 | 1.47 | 0.364 | 0.721 |
| hsa-mir-31 | 2090 | ± | 961 | 2973 | ± | 1225 | 1.42 | 0.364 | 0.721 |
| hsa-mir-4659b | 5 | ± | 5 | 0 | ± | 0 | 0.00 | 0.363 | 0.721 |
| chr14_79353108_79353217_+ | 5 | ± | 5 | 0 | ± | 0 | 0.00 | 0.362 | 0.721 |
| hsa-mir-139 | 4907 | ± | 597 | 3495 | ± | 629 | -1.40 | 0.357 | 0.721 |
| hsa-mir-130b | 3194 | ± | 1167 | 2250 | ± | 577 | -1.42 | 0.360 | 0.721 |
| chr6_133138407_133138519_+ | 22 | ± | 7 | 8 | ± | 8 | -2.77 | 0.355 | 0.721 |
| chr2_219206612_219206723_+ | 18 | ± | 9 | 4 | ± | 4 | -4.24 | 0.361 | 0.721 |
| hsa-mir-1307 | 57 | ± | 14 | 35 | ± | 12 | -1.65 | 0.367 | 0.723 |
| hsa-mir-196b | 49 | ± | 8 | 75 | ± | 16 | 1.52 | 0.382 | 0.737 |
| hsa-mir-146b | 709 | ± | 166 | 1001 | ± | 264 | 1.41 | 0.404 | 0.737 |
| hsa-mir-374b | 1031 | ± | 350 | 1452 | ± | 387 | 1.41 | 0.399 | 0.737 |
| hsa-mir-340 | 4327 | ± | 246 | 5936 | ± | 968 | 1.37 | 0.387 | 0.737 |
| hsa-mir-142 | 57195 | ± | 7009 | 74478 | ± | 11778 | 1.30 | 0.396 | 0.737 |
| hsa-mir-2964a | 5 | ± | 5 | 0 | ± | 0 | 0.00 | 0.387 | 0.737 |
| chrX_153209586_153209699_- | 5 | ± | 5 | 0 | ± | 0 | 0.00 | 0.387 | 0.737 |
| hsa-mir-148b | 3376 | ± | 99 | 2433 | ± | 90 | -1.39 | 0.389 | 0.737 |
| hsa-mir-641 | 121 | ± | 13 | 82 | ± | 9 | -1.48 | 0.377 | 0.737 |
| hsa-mir-4326 | 84 | ± | 14 | 56 | ± | 17 | -1.50 | 0.402 | 0.737 |
| hsa-mir-92b | 58 | ± | 12 | 36 | ± | 10 | -1.63 | 0.379 | 0.737 |
| hsa-mir-3074 | 28 | ± | 3 | 14 | ± | 8 | -2.07 | 0.398 | 0.737 |
| chr16_29816013_29816124_+ | 23 | ± | 11 | 10 | ± | 7 | -2.35 | 0.405 | 0.737 |
| hsa-mir-431 | 22 | ± | 11 | 9 | ± | 6 | -2.52 | 0.394 | 0.737 |
| chr2_219144818_219144940_- | 18 | ± | 8 | 5 | ± | 5 | -3.37 | 0.392 | 0.737 |
| hsa-mir-3657 | 5 | ± | 5 | 0 | ± | 0 | 0.00 | 0.411 | 0.743 |
| hsa-mir-185 | 26 | ± | 3 | 13 | ± | 8 | -2.08 | 0.412 | 0.743 |
| hsa-mir-181a-2 | 112 | ± | 21 | 157 | ± | 34 | 1.41 | 0.417 | 0.748 |
| hsa-mir-181a-1 | 552 | ± | 198 | 768 | ± | 63 | 1.39 | 0.431 | 0.751 |
| hsa-mir-29c | 18494 | ± | 4337 | 23871 | ± | 4947 | 1.29 | 0.439 | 0.751 |
| hsa-mir-3609 | 5 | ± | 5 | 0 | ± | 0 | 0.00 | 0.434 | 0.751 |
| chr16_30913808_30913918_- | 5 | ± | 5 | 0 | ± | 0 | 0.00 | 0.434 | 0.751 |
| chr10_16636294_16636404_- | 5 | ± | 5 | 0 | ± | 0 | 0.00 | 0.434 | 0.751 |
| chr1_7253527_7253637_+ | 5 | ± | 5 | 0 | ± | 0 | 0.00 | 0.434 | 0.751 |
| chr11_93463653_93463762_- | 201 | ± | 84 | 145 | ± | 54 | -1.39 | 0.439 | 0.751 |
| hsa-mir-7-2 | 35 | ± | 3 | 20 | ± | 9 | -1.77 | 0.430 | 0.751 |
| chr16_88535323_88535432_+ | 24 | ± | 7 | 12 | ± | 7 | -2.10 | 0.438 | 0.751 |
| chr3_122692516_122692627_- | 10 | ± | 7 | 3 | ± | 3 | -3.40 | 0.432 | 0.751 |
| hsa-mir-320a | 140 | ± | 92 | 192 | ± | 66 | 1.37 | 0.447 | 0.760 |
| hsa-mir-660 | 3112 | ± | 582 | 2346 | ± | 544 | -1.33 | 0.460 | 0.779 |
| hsa-mir-1468 | 11 | ± | 6 | 3 | ± | 3 | -3.12 | 0.462 | 0.780 |
| chr15_69109017_69109125_- | 14 | ± | 6 | 25 | ± | 9 | 1.76 | 0.469 | 0.785 |
| hsa-mir-324 | 587 | ± | 224 | 433 | ± | 194 | -1.36 | 0.469 | 0.785 |
| hsa-mir-3120 | 46 | ± | 9 | 30 | ± | 13 | -1.55 | 0.472 | 0.786 |
| hsa-mir-579 | 46 | ± | 12 | 30 | ± | 8 | -1.53 | 0.488 | 0.807 |
| hsa-mir-769 | 92 | ± | 47 | 124 | ± | 31 | 1.34 | 0.491 | 0.809 |
| hsa-mir-1271 | 211 | ± | 21 | 281 | ± | 72 | 1.33 | 0.494 | 0.811 |
| hsa-mir-425 | 52396 | ± | 6062 | 64786 | ± | 10356 | 1.24 | 0.497 | 0.813 |
| hsa-mir-545 | 49 | ± | 9 | 33 | ± | 10 | -1.48 | 0.500 | 0.813 |
| hsa-mir-629 | 93 | ± | 6 | 123 | ± | 26 | 1.32 | 0.514 | 0.833 |
| hsa-mir-3191 | 6 | ± | 6 | 0 | ± | 0 | 0.00 | 0.525 | 0.843 |
| hsa-mir-22 | 1885 | ± | 442 | 1466 | ± | 636 | -1.29 | 0.525 | 0.843 |
| hsa-mir-3912 | 11 | ± | 5 | 6 | ± | 6 | -1.90 | 0.527 | 0.843 |
| hsa-mir-877 | 10 | ± | 7 | 19 | ± | 13 | 1.79 | 0.536 | 0.846 |
| hsa-mir-19a | 100903 | ± | 19073 | 83287 | ± | 8361 | -1.21 | 0.533 | 0.846 |
| hsa-mir-144 | 779 | ± | 133 | 604 | ± | 174 | -1.29 | 0.539 | 0.846 |
| hsa-mir-377 | 11 | ± | 8 | 2 | ± | 2 | -4.58 | 0.536 | 0.846 |
| hsa-mir-106b | 1598 | ± | 397 | 2024 | ± | 502 | 1.27 | 0.551 | 0.848 |
| hsa-mir-20a | 8212 | ± | 2880 | 10117 | ± | 2480 | 1.23 | 0.549 | 0.848 |
| hsa-mir-30d | 11370 | ± | 2097 | 9238 | ± | 725 | -1.23 | 0.545 | 0.848 |
| hsa-mir-1-1 | 14 | ± | 6 | 6 | ± | 6 | -2.57 | 0.551 | 0.848 |
| hsa-mir-597 | 15 | ± | 6 | 6 | ± | 6 | -2.62 | 0.544 | 0.848 |
| hsa-mir-181b-2 | 32 | ± | 5 | 44 | ± | 6 | 1.38 | 0.575 | 0.850 |
| hsa-mir-2277 | 34 | ± | 12 | 46 | ± | 9 | 1.37 | 0.573 | 0.850 |
| hsa-mir-16-2 | 516 | ± | 129 | 655 | ± | 112 | 1.27 | 0.572 | 0.850 |
| hsa-mir-505 | 1493 | ± | 114 | 1871 | ± | 98 | 1.25 | 0.571 | 0.850 |
| hsa-mir-215 | 7 | ± | 7 | 0 | ± | 0 | 0.00 | 0.568 | 0.850 |
| hsa-mir-15b | 29396 | ± | 5443 | 24515 | ± | 3793 | -1.20 | 0.575 | 0.850 |
| hsa-mir-30b | 11597 | ± | 2314 | 9569 | ± | 1210 | -1.21 | 0.575 | 0.850 |
| hsa-mir-624 | 53 | ± | 8 | 39 | ± | 12 | -1.37 | 0.571 | 0.850 |
| hsa-mir-1248 | 10 | ± | 7 | 6 | ± | 6 | -1.86 | 0.576 | 0.850 |
| chr22_26968258_26968383_- | 18 | ± | 12 | 9 | ± | 5 | -2.00 | 0.565 | 0.850 |
| hsa-let-7g | 25097 | ± | 5594 | 30044 | ± | 3319 | 1.20 | 0.579 | 0.852 |
| hsa-mir-151 | 10 | ± | 6 | 5 | ± | 5 | -2.04 | 0.586 | 0.857 |
| chr10_124176475_124176587_- | 10 | ± | 7 | 17 | ± | 9 | 1.70 | 0.590 | 0.861 |
| hsa-mir-671 | 272 | ± | 25 | 218 | ± | 55 | -1.25 | 0.595 | 0.864 |
| chr5_138614450_138614564_+ | 21 | ± | 9 | 30 | ± | 9 | 1.44 | 0.598 | 0.865 |
| hsa-mir-33a | 1705 | ± | 342 | 2096 | ± | 373 | 1.23 | 0.601 | 0.866 |
| hsa-mir-3605 | 41 | ± | 3 | 53 | ± | 13 | 1.31 | 0.609 | 0.870 |
| hsa-mir-93 | 3704 | ± | 832 | 4468 | ± | 1580 | 1.21 | 0.614 | 0.870 |
| hsa-mir-363 | 231 | ± | 51 | 187 | ± | 28 | -1.24 | 0.612 | 0.870 |
| hsa-mir-33b | 68 | ± | 16 | 53 | ± | 15 | -1.28 | 0.617 | 0.870 |
| hsa-mir-1226 | 65 | ± | 20 | 50 | ± | 5 | -1.29 | 0.618 | 0.870 |
| chr3_127305928_127306044_- | 18 | ± | 8 | 10 | ± | 7 | -1.78 | 0.611 | 0.870 |
| hsa-let-7i | 1351 | ± | 281 | 1646 | ± | 439 | 1.22 | 0.622 | 0.872 |
| hsa-mir-145 | 9048 | ± | 2299 | 7627 | ± | 1554 | -1.19 | 0.624 | 0.872 |
| hsa-mir-20b | 1816 | ± | 298 | 2196 | ± | 217 | 1.21 | 0.629 | 0.872 |
| hsa-mir-486 | 7 | ± | 5 | 0 | ± | 0 | 0.00 | 0.629 | 0.872 |
| chr2_74766424_74766533_- | 124 | ± | 33 | 101 | ± | 11 | -1.23 | 0.633 | 0.875 |
| hsa-mir-1296 | 12 | ± | 8 | 5 | ± | 5 | -2.26 | 0.638 | 0.878 |
| hsa-mir-628 | 444 | ± | 73 | 539 | ± | 49 | 1.21 | 0.648 | 0.884 |
| hsa-mir-25 | 8347 | ± | 870 | 7105 | ± | 1185 | -1.17 | 0.646 | 0.884 |
| hsa-mir-1301 | 205 | ± | 48 | 171 | ± | 53 | -1.19 | 0.672 | 0.914 |
| hsa-mir-182 | 528 | ± | 98 | 627 | ± | 117 | 1.19 | 0.680 | 0.922 |
| hsa-mir-598 | 633 | ± | 129 | 534 | ± | 74 | -1.19 | 0.685 | 0.925 |
| chr3_49843579_49843688_- | 95 | ± | 7 | 79 | ± | 9 | -1.20 | 0.692 | 0.927 |
| chr4_3230264_3230373_+ | 14 | ± | 6 | 8 | ± | 5 | -1.73 | 0.690 | 0.927 |
| hsa-mir-24-2 | 1568 | ± | 227 | 1831 | ± | 269 | 1.17 | 0.695 | 0.929 |
| hsa-mir-504 | 24 | ± | 12 | 30 | ± | 12 | 1.29 | 0.704 | 0.933 |
| hsa-mir-26a-2 | 18 | ± | 9 | 12 | ± | 8 | -1.49 | 0.703 | 0.933 |
| hsa-mir-491 | 33 | ± | 4 | 41 | ± | 17 | 1.24 | 0.719 | 0.940 |
| hsa-mir-17 | 6864 | ± | 1235 | 7784 | ± | 879 | 1.13 | 0.722 | 0.940 |
| hsa-mir-30e | 11503 | ± | 1436 | 13023 | ± | 1994 | 1.13 | 0.716 | 0.940 |
| hsa-mir-23a | 94923 | ± | 12853 | 85256 | ± | 10185 | -1.11 | 0.727 | 0.940 |
| chr2_25551539_25551648_- | 161 | ± | 35 | 139 | ± | 43 | -1.16 | 0.724 | 0.940 |
| hsa-let-7f-2 | 45 | ± | 7 | 37 | ± | 16 | -1.24 | 0.715 | 0.940 |
| hsa-let-7c | 40 | ± | 9 | 32 | ± | 21 | -1.24 | 0.722 | 0.940 |
| hsa-mir-301b | 762 | ± | 93 | 875 | ± | 231 | 1.15 | 0.738 | 0.942 |
| hsa-mir-125a | 19862 | ± | 3277 | 17767 | ± | 2363 | -1.12 | 0.736 | 0.942 |
| hsa-mir-29b-2 | 215 | ± | 19 | 186 | ± | 9 | -1.16 | 0.732 | 0.942 |
| hsa-mir-16-1 | 23 | ± | 6 | 17 | ± | 8 | -1.34 | 0.738 | 0.942 |
| hsa-mir-451 | 53820 | ± | 7087 | 59635 | ± | 13404 | 1.11 | 0.743 | 0.943 |
| hsa-mir-744 | 546 | ± | 54 | 476 | ± | 32 | -1.15 | 0.744 | 0.943 |
| hsa-mir-106a | 16 | ± | 7 | 11 | ± | 7 | -1.44 | 0.748 | 0.943 |
| hsa-mir-99a | 926 | ± | 94 | 1046 | ± | 107 | 1.13 | 0.766 | 0.957 |
| hsa-mir-2110 | 35 | ± | 6 | 29 | ± | 8 | -1.22 | 0.764 | 0.957 |
| hsa-mir-1250 | 15 | ± | 7 | 11 | ± | 6 | -1.43 | 0.765 | 0.957 |
| hsa-mir-339 | 1199 | ± | 319 | 1350 | ± | 208 | 1.13 | 0.769 | 0.957 |
| hsa-mir-103a-1 | 15 | ± | 6 | 10 | ± | 6 | -1.42 | 0.775 | 0.961 |
| hsa-mir-199a-1 | 9 | ± | 6 | 12 | ± | 9 | 1.36 | 0.791 | 0.962 |
| hsa-let-7a-1 | 12 | ± | 7 | 15 | ± | 7 | 1.29 | 0.800 | 0.962 |
| hsa-mir-103a-2 | 59 | ± | 6 | 67 | ± | 10 | 1.14 | 0.788 | 0.962 |
| chr22_43011305_43011415_+ | 65 | ± | 18 | 72 | ± | 23 | 1.12 | 0.810 | 0.962 |
| hsa-mir-301a | 27989 | ± | 3546 | 30330 | ± | 3747 | 1.08 | 0.804 | 0.962 |
| hsa-mir-4701 | 9 | ± | 6 | 0 | ± | 0 | 0.00 | 0.798 | 0.962 |
| chr7_1987324_1987435_- | 9 | ± | 6 | 0 | ± | 0 | 0.00 | 0.783 | 0.962 |
| chr12_113729321_113729433_+ | 9 | ± | 6 | 0 | ± | 0 | 0.00 | 0.783 | 0.962 |
| hsa-mir-484 | 31962 | ± | 3441 | 29616 | ± | 3121 | -1.08 | 0.812 | 0.962 |
| hsa-let-7b | 1393 | ± | 332 | 1266 | ± | 325 | -1.10 | 0.811 | 0.962 |
| hsa-mir-210 | 1116 | ± | 538 | 1007 | ± | 294 | -1.11 | 0.799 | 0.962 |
| hsa-mir-24-1 | 37 | ± | 4 | 31 | ± | 8 | -1.18 | 0.794 | 0.962 |
| hsa-mir-18b | 29 | ± | 9 | 25 | ± | 7 | -1.19 | 0.812 | 0.962 |
| hsa-mir-187 | 30 | ± | 14 | 25 | ± | 11 | -1.20 | 0.798 | 0.962 |
| hsa-mir-574 | 6052 | ± | 1964 | 6569 | ± | 1384 | 1.09 | 0.819 | 0.967 |
| hsa-mir-362 | 338 | ± | 43 | 371 | ± | 37 | 1.10 | 0.824 | 0.968 |
| hsa-mir-3620 | 10 | ± | 6 | 0 | ± | 0 | 0.00 | 0.825 | 0.968 |
| hsa-mir-590 | 6774 | ± | 1189 | 7281 | ± | 728 | 1.07 | 0.839 | 0.975 |
| hsa-mir-1185-2 | 10 | ± | 6 | 0 | ± | 0 | 0.00 | 0.837 | 0.975 |
| chr17_75675439_75675547_+ | 11 | ± | 7 | 9 | ± | 6 | -1.23 | 0.834 | 0.975 |
| hsa-mir-1537 | 18 | ± | 10 | 21 | ± | 16 | 1.15 | 0.861 | 0.985 |
| hsa-mir-26a-1 | 116 | ± | 29 | 125 | ± | 18 | 1.08 | 0.858 | 0.985 |
| hsa-mir-27a | 31921 | ± | 1446 | 30160 | ± | 6043 | -1.06 | 0.860 | 0.985 |
| hsa-mir-3064 | 28 | ± | 6 | 25 | ± | 11 | -1.15 | 0.852 | 0.985 |
| hsa-mir-92a-1 | 13 | ± | 13 | 10 | ± | 7 | -1.28 | 0.861 | 0.985 |
| chr3_52427940_52428049_+ | 13 | ± | 8 | 16 | ± | 6 | 1.18 | 0.868 | 0.987 |
| hsa-mir-19b-1 | 173 | ± | 21 | 161 | ± | 22 | -1.07 | 0.868 | 0.987 |
| chr12_6690620_6690732_- | 0 | ± | 0 | 12 | ± | 7 | 0.00 | 0.878 | 0.995 |
| hsa-mir-98 | 935 | ± | 281 | 989 | ± | 202 | 1.06 | 0.892 | 1.000 |
| hsa-mir-4677 | 32 | ± | 6 | 34 | ± | 11 | 1.06 | 0.930 | 1.000 |
| chr15_83424731_83424839_+ | 1702 | ± | 128 | 1788 | ± | 76 | 1.05 | 0.902 | 1.000 |
| hsa-mir-100 | 96 | ± | 37 | 100 | ± | 31 | 1.05 | 0.914 | 1.000 |
| hsa-mir-501 | 52 | ± | 14 | 54 | ± | 6 | 1.05 | 0.929 | 1.000 |
| hsa-mir-7-1 | 2119 | ± | 336 | 2215 | ± | 568 | 1.05 | 0.909 | 1.000 |
| hsa-mir-181b-1 | 97 | ± | 23 | 102 | ± | 11 | 1.05 | 0.919 | 1.000 |
| chr9_19063627_19063737_- | 131 | ± | 74 | 136 | ± | 33 | 1.04 | 0.918 | 1.000 |
| hsa-mir-188 | 254 | ± | 58 | 263 | ± | 21 | 1.04 | 0.932 | 1.000 |
| hsa-mir-345 | 285 | ± | 74 | 294 | ± | 50 | 1.03 | 0.940 | 1.000 |
| hsa-mir-361 | 2658 | ± | 408 | 2736 | ± | 151 | 1.03 | 0.940 | 1.000 |
| hsa-mir-181c | 1368 | ± | 189 | 1402 | ± | 240 | 1.02 | 0.952 | 1.000 |
| hsa-mir-15a | 2345 | ± | 405 | 2352 | ± | 332 | 1.00 | 0.994 | 1.000 |
| hsa-mir-3615 | 156 | ± | 36 | 156 | ± | 57 | 1.00 | 0.998 | 1.000 |
| hsa-mir-938 | 0 | ± | 0 | 4 | ± | 4 | 0.00 | 1.000 | 1.000 |
| hsa-mir-873 | 0 | ± | 0 | 10 | ± | 10 | 0.00 | 1.000 | 1.000 |
| hsa-mir-770 | 17 | ± | 11 | 0 | ± | 0 | 0.00 | 1.000 | 1.000 |
| hsa-mir-665 | 0 | ± | 0 | 4 | ± | 4 | 0.00 | 1.000 | 1.000 |
| hsa-mir-655 | 10 | ± | 6 | 0 | ± | 0 | 0.00 | 1.000 | 1.000 |
| hsa-mir-4645 | 12 | ± | 5 | 0 | ± | 0 | 0.00 | 1.000 | 1.000 |
| hsa-mir-455 | 0 | ± | 0 | 8 | ± | 5 | 0.00 | 1.000 | 1.000 |
| hsa-mir-4423 | 0 | ± | 0 | 8 | ± | 8 | 0.00 | 1.000 | 1.000 |
| hsa-mir-3667 | 0 | ± | 0 | 5 | ± | 5 | 0.00 | 1.000 | 1.000 |
| hsa-mir-3617 | 0 | ± | 0 | 6 | ± | 6 | 0.00 | 1.000 | 1.000 |
| hsa-mir-3136 | 17 | ± | 7 | 0 | ± | 0 | 0.00 | 1.000 | 1.000 |
| hsa-mir-196a-1 | 0 | ± | 0 | 8 | ± | 5 | 0.00 | 1.000 | 1.000 |
| hsa-mir-1277 | 17 | ± | 7 | 0 | ± | 0 | 0.00 | 1.000 | 1.000 |
| chr8_99405876_99405985_- | 0 | ± | 0 | 2 | ± | 2 | 0.00 | 1.000 | 1.000 |
| chr6_70671956_70672069_+ | 0 | ± | 0 | 6 | ± | 6 | 0.00 | 1.000 | 1.000 |
| chr22_20052684_20052792_+ | 0 | ± | 0 | 4 | ± | 4 | 0.00 | 1.000 | 1.000 |
| chr20_2635763_2635872_+ | 0 | ± | 0 | 5 | ± | 5 | 0.00 | 1.000 | 1.000 |
| chr17_76136804_76136912_+ | 0 | ± | 0 | 12 | ± | 8 | 0.00 | 0.896 | 1.000 |
| chr11_64109291_64109400_+ | 0 | ± | 0 | 5 | ± | 5 | 0.00 | 1.000 | 1.000 |
| chr11_14521302_14521408_- | 0 | ± | 0 | 6 | ± | 6 | 0.00 | 1.000 | 1.000 |
| chr10_114468945_114469054_+ | 0 | ± | 0 | 2 | ± | 2 | 0.00 | 1.000 | 1.000 |
| chr1_203698737_203698850_+ | 0 | ± | 0 | 3 | ± | 3 | 0.00 | 1.000 | 1.000 |
| chr1_175937506_175937664_- | 13 | ± | 8 | 0 | ± | 0 | 0.00 | 1.000 | 1.000 |
| hsa-mir-148a | 6762 | ± | 731 | 6663 | ± | 1717 | -1.01 | 0.967 | 1.000 |
| hsa-mir-378 | 280 | ± | 56 | 269 | ± | 69 | -1.04 | 0.924 | 1.000 |
| hsa-mir-500a | 39 | ± | 10 | 37 | ± | 10 | -1.05 | 0.942 | 1.000 |
| hsa-let-7d | 5995 | ± | 2790 | 5700 | ± | 1852 | -1.05 | 0.888 | 1.000 |
| hsa-mir-155 | 466 | ± | 80 | 440 | ± | 63 | -1.06 | 0.892 | 1.000 |
| hsa-mir-643 | 17 | ± | 7 | 15 | ± | 7 | -1.12 | 0.915 | 1.000 |
