# Supplementary material for: High-Throughput Sequencing of microRNAs in Peripheral Blood Mononuclear Cells: Identification of Potential Weight Loss Biomarkers
Source: PLoS One. 2013 Jan 15;8(1):e54319. doi: 10.1371/journal.pone.0054319 (PMC3545952; doi:10.1371/journal.pone.0054319)
Supplement: Table S2 — miRNA clusters (according to miRBase) upregulated in peripheral blood cells of non-responders to the low-calorie diet when compared to the responders, categorized by chromosomal location. (DOC) [file pone.0054319.s002.doc]

**Supplementary table 2.** miRNA clusters (according to miRBase) upregulated in peripheral blood cells of non-responders to the low-calorie diet when compared to the responders, categorized by chromosomal location. The data report the mean of the number of transcripts sequenced in each group ± SEM and the fold change between groups. Student's t-test has been used to compare responders and non-responders.

| **miRNA** | **Accession**  **number** | **Chromosome** | **Start** | **End** | **Responders**  **Mean ±SEM** | | | **Non-responders**  **Mean ±SEM** | | | **Fold change**  **Mean** | **P-value**  **Mean** |
| --- | --- | --- | --- | --- | --- | --- | --- | --- | --- | --- | --- | --- |
| hsa-mir-542 | MI0003686 | X | 133675371 | 133675467 | 1881 | ± | 103 | 4121 | ± | 785 | 2.71 | 0.14 |
| hsa-mir-424 | MI0001446 |  | 133680644 | 133680741 |  |  |  |  |  |  |  |  |
| hsa-mir-450b | [MI0005531](http://www.mirbase.org/cgi-bin/mirna_entry.pl?acc=MI0005531) |  | 133674215 | 133674292 |  |  |  |  |  |  |  |  |
| hsa-mir-223 | MI0000300 | X | 65238712 | 65238821 | 3108550 | ± | 533782 | 7618398 | ± | 2590465 | 2.45 | 0.13 |
| hsa-mir-532 | MI0003205 | X | 49767754 | 49767844 | 1605 | ± | 530 | 4145 | ± | 622 | 2.58 | **0.01** |
| hsa-mir-371b | MI0000779 | 19 | 54290929 | 54290995 | 5 | ± | 5 | 27 | ± | 11 | 5.95 | 0.10 |
| hsa-mir-935 | MI0005757 | 18 | 54485561 | 54485651 | 18 | ± | 11 | 125 | ± | 44 | 7.09 | **0.04** |
| hsa-mir-338 | MI0000814 | 17 | 79099683 | 79099749 | 1537 | ± | 201 | 3319 | ± | 698 | 2.99 | 0.12 |
| hsa-mir-3614 | MI0016004 | 17 | 54968631 | 54968716 |  |  |  |  |  |  |  |  |
| hsa-mir-342 | MI0000805 | 14 | 100575992 | 100576090 | 135235 | ± | 29150 | 281525 | ± | 60554 | 2.08 | 0.06 |
| hsa-mir-618 | MI0003632 | 12 | 81329515 | 81329612 | 10 | ± | 6 | 24 | ± | 11 | 2.43 | 0.30 |
| hsa-mir-199b | MI0000282 | 9 | 131007000 | 131007109 | 850 | ± | 41 | 2100 | ± | 301 | 2.27 | **0.02** |
| hsa-mir-204 | MI0000284 |  | 73424891 | 73425000 |  |  |  |  |  |  |  |  |
| hsa-mir-29b-1 | MI0000105 | 7 | 130562218 | 130562298 | 40 | ± | 6 | 105 | ± | 17 | 4.72 | 0.07 |
| hsa-mir-183 | MI0000273 |  | 129414745 | 129414854 |  |  |  |  |  |  |  |  |
| hsa-mir-96 | MI0000098 |  | 129414532 | 129414609 |  |  |  |  |  |  |  |  |
| hsa-mir-874 | MI0005532 | 5 | 136983261 | 136983338 | 127 | ± | 10 | 328 | ± | 62 | 2.56 | 0.10 |
| hsa-mir-3607 | MI0015997 |  | 85916314 | 85916392 |  |  |  |  |  |  |  |  |
| hsa-mir-582 | MI0003589 |  | 58999432 | 58999529 |  |  |  |  |  |  |  |  |
| hsa-mir-2115 | MI0010634 | 3 | 48357850 | 48357949 | 5 | ± | 2 | 20 | ± | 5 | 3.59 | 0.10 |
| hsa-mir-138-1 | MI0000476 |  | 44155704 | 44155802 |  |  |  |  |  |  |  |  |
