# Supplementary material for: High-Throughput Sequencing of microRNAs in Peripheral Blood Mononuclear Cells: Identification of Potential Weight Loss Biomarkers
Source: PLoS One. 2013 Jan 15;8(1):e54319. doi: 10.1371/journal.pone.0054319 (PMC3545952; doi:10.1371/journal.pone.0054319)
Supplement: Table S3 — miRNA clusters (according to miRBase) downregulated in peripheral blood cells of non-responders to the low-calorie diet when compared to the responders, categorized by chromosomal location. (DOC) [file pone.0054319.s003.doc]

**Supplementary table 3.** miRNA clusters (according to miRBase) downregulated in peripheral blood cells of non-responders to the low-calorie diet when compared to the responders, categorized by chromosomal location. The data report the mean of the number of transcripts sequenced in each group ± SEM and the fold change between groups. Student's t-test has been used to compare responders and non-responders.

| **miRNA** | **Accession number** | **Chromosome** | **Start** | **End** | **Responders**  **Mean ±SEM** | | | **Non-responders**  **Mean ±SEM** | | | **Fold change**  **Mean** | **P-value**  **Mean** |
| --- | --- | --- | --- | --- | --- | --- | --- | --- | --- | --- | --- | --- |
| hsa-mir-452 | MI0001733 | X | 151128100 | 151128184 | 441 | ± | 115 | 119 | ± | 12 | -3.11 | 0.08 |
| hsa-mir-224 | MI0000301 |  | 151127050 | 151127130 |  |  |  |  |  |  |  |  |
| hsa-mir-503 | MI0003188 | X | 133680358 | 133680428 | 56 | ± | 18 | 15 | ± | 10 | -3.61 | 0.09 |
| hsa-mir-19b-2 | MI0000075 | X | 133303701 | 133303796 | 28 | ± | 8 | 4 | ± | 4 | -7.31 | **0.03** |
| hsa-mir-766 | MI0003836 | X | 118780701 | 118780811 | 54 | ± | 4 | 14 | ± | 9 | -3.80 | **< 0.01** |
| hsa-mir-1468 | MI0003782 | X | 63005882 | 63005967 | 11 | ± | 6 | 3 | ± | 3 | -3.12 | 0.35 |
| hsa-mir-502 | MI0003186 | X | 49779206 | 49779291 | 30 | ± | 13 | 14 | ± | 14 | -2.22 | 0.40 |
| hsa-mir-185 | MI0000482 | 22 | 20020662 | 20020743 | 26 | ± | 3 | 13 | ± | 8 | -2.08 | 0.15 |
| hsa-mir-1-1 | MI0000651 | 20 | 61151513 | 61151583 | 14 | ± | 6 | 6 | ± | 6 | -2.57 | 0.31 |
| hsa-let-7e | MI0000066 | 19 | 52196039 | 52196117 | 667 | ± | 144 | 321 | ± | 34 | -2.21 | 0.21 |
| hsa-mir-99b | MI0000746 |  | 52195865 | 52195934 |  |  |  |  |  |  |  |  |
| hsa-mir-3940 | MI0016597 |  | 6416421 | 6416522 |  |  |  |  |  |  |  |  |
| hsa-mir-656 | MI0003678 | 14 | 101533061 | 101533138 | 712 | ± | 62 | 261 | ± | 23 | -3.55 | 0.18 |
| hsa-mir-410 | MI0002465 |  | 101532249 | 101532328 |  |  |  |  |  |  |  |  |
| hsa-mir-369 | MI0000777 |  | 101531935 | 101532004 |  |  |  |  |  |  |  |  |
| hsa-mir-409 | MI0001735 |  | 101531637 | 101531715 |  |  |  |  |  |  |  |  |
| hsa-mir-377 | MI0000785 |  | 101528387 | 101528455 |  |  |  |  |  |  |  |  |
| hsa-mir-496 | MI0003136 |  | 101526910 | 101527011 |  |  |  |  |  |  |  |  |
| hsa-mir-154 | MI0000480 |  | 101526092 | 101526175 |  |  |  |  |  |  |  |  |
| hsa-mir-485 | MI0002469 |  | 101521756 | 101521828 |  |  |  |  |  |  |  |  |
| hsa-mir-668 | MI0003761 |  | 101521595 | 101521660 |  |  |  |  |  |  |  |  |
| hsa-mir-134 | MI0000474 |  | 101521024 | 101521096 |  |  |  |  |  |  |  |  |
| hsa-mir-382 | MI0000790 |  | 101520643 | 101520718 |  |  |  |  |  |  |  |  |
| hsa-mir-487a | MI0002471 |  | 101518783 | 101518862 |  |  |  |  |  |  |  |  |
| hsa-mir-539 | MI0003514 |  | 101513658 | 101513735 |  |  |  |  |  |  |  |  |
| hsa-mir-487b | MI0003530 |  | 101512792 | 101498401 |  |  |  |  |  |  |  |  |
| hsa-mir-381 | MI0000789 |  | 101512257 | 101512331 |  |  |  |  |  |  |  |  |
| hsa-mir-1185-1 | MI0003844 |  | 101509314 | 101509399 |  |  |  |  |  |  |  |  |
| hsa-mir-376a-1 | MI0000784 |  | 101507119 | 101507186 |  |  |  |  |  |  |  |  |
| hsa-mir-376b | MI0002466 |  | 101506773 | 101506872 |  |  |  |  |  |  |  |  |
| hsa-mir-654 | MI0003676 |  | 101506556 | 101506636 |  |  |  |  |  |  |  |  |
| hsa-mir-376a-2 | MI0003529 |  | 101506406 | 101506485 |  |  |  |  |  |  |  |  |
| hsa-mir-376c | MI0000776 |  | 101506027 | 101506092 |  |  |  |  |  |  |  |  |
| hsa-mir-543 | MI0005565 | 14 | 101498324 | 101498401 | 585 | ± | 63 | 205 | ± | 36 | -3.07 | 0.08 |
| hsa-mir-494 | MI0003134 |  | 101495971 | 101496051 |  |  |  |  |  |  |  |  |
| hsa-mir-758 | MI0003757 |  | 101492357 | 101492444 |  |  |  |  |  |  |  |  |
| hsa-mir-323 | MI0000807 |  | 101492069 | 101492154 |  |  |  |  |  |  |  |  |
| hsa-mir-299 | MI0000744 |  | 101490131 | 101490193 |  |  |  |  |  |  |  |  |
| hsa-mir-411 | [MI0003675](http://www.mirbase.org/cgi-bin/mirna_entry.pl?acc=MI0003675) |  | 101489662 | 101489757 |  |  |  |  |  |  |  |  |
| hsa-mir-379 | MI0000787 |  | 101488403 | 101488469 |  |  |  |  |  |  |  |  |
| hsa-mir-370 | MI0000778 | 14 | 101377476 | 101377550 | 367 | ± | 38 | 129 | ± | 24 | -3.66 | 0.10 |
| hsa-mir-136 | MI0000475 |  | 101351039 | 101351120 |  |  |  |  |  |  |  |  |
| hsa-mir-432 | MI0003133 |  | 101350820 | 101350913 |  |  |  |  |  |  |  |  |
| hsa-mir-127 | MI0000472 |  | 101349316 | 101349412 |  |  |  |  |  |  |  |  |
| hsa-mir-433 | MI0001723 |  | 101348223 | 101348315 |  |  |  |  |  |  |  |  |
| hsa-mir-431 | MI0001721 |  | 101347344 | 101347457 |  |  |  |  |  |  |  |  |
| hsa-mir-337 | MI0000806 |  | 101340830 | 101340922 |  |  |  |  |  |  |  |  |
| hsa-mir-493 | MI0003132 |  | 101335397 | 101335485 |  |  |  |  |  |  |  |  |
| hsa-mir-18a | [MI0000072](http://www.mirbase.org/cgi-bin/mirna_entry.pl?acc=MI0000072) | 13 | 92003005 | 92003075 | 2452 | ± | 956 | 1187 | ± | 182 | -2.07 | 0.23 |
| hsa-mir-1296 | MI0003780 | 10 | 65132717 | 65132808 | 12 | ± | 8 | 5 | ± | 5 | -2.26 | 0.49 |
| hsa-mir-3074 | MI0014181 | 9 | 97848296 | 97848376 | 842 | ± | 195 | 250 | ± | 77 | -2.74 | 0.08 |
| hsa-mir-27b | MI0000440 |  | 97847727 | 97847823 |  |  |  |  |  |  |  |  |
| hsa-mir-4662a | MI0017290 | 8 | 125834227 | 125834293 | 16 | ± | 6 | 5 | ± | 2 | -3.72 | 0.23 |
| hsa-mir-597 | MI0003609 |  | 9599182 | 9599278 |  |  |  |  |  |  |  |  |
| hsa-mir-589 | [MI0003599](http://www.mirbase.org/cgi-bin/mirna_entry.pl?acc=MI0003599) | 7 | 5535450 | 5535548 | 93 | ± | 8 | 38 | ± | 11 | -2.46 | **<0.01** |
| hsa-mir-1229 | [MI0006319](http://www.mirbase.org/cgi-bin/mirna_entry.pl?acc=MI0006319) | 5 | 179225278 | 179225346 | 67 | ± | 16 | 27 | ± | 13 | -2.51 | 0.09 |
| hsa-mir-95 | MI0000097 | 4 | 8007028 | 8007108 | 9 | ± | 6 | 3 | ± | 3 | -3.38 | 0.34 |
| hsa-mir-570 | MI0003577 | 3 | 195426272 | 195426368 | 93 | ± | 11 | 42 | ± | 14 | -2.28 | 0.08 |
| hsa-mir-551b | MI0003575 |  | 168269642 | 168269737 |  |  |  |  |  |  |  |  |
