# Supplementary material for: High-Throughput Sequencing of microRNAs in Peripheral Blood Mononuclear Cells: Identification of Potential Weight Loss Biomarkers
Source: PLoS One. 2013 Jan 15;8(1):e54319. doi: 10.1371/journal.pone.0054319 (PMC3545952; doi:10.1371/journal.pone.0054319)
Supplement: Table S4 — The most expressed miRNA transcripts in PBMC cells, based on read counts, and the chromosome where they are located. (DOC) [file pone.0054319.s004.doc]

**Supplementary table 4.** The most expressed miRNA transcripts in PBMC cells, based on read counts, and the chromosome where they are located.

| **miRNA Transcripts Location (**source **Pubmed)** |
| --- |
| **mir-223** 5363474 X |
| **mir-150** 464354 19q13.33 |
| **mir-126** 359563 9q34.3 |
| **mir-21** 232550 17q23.1 |
| **mir-342** 208380 14q32.2 |
| **mir-191** 136849 3p21.31 |
| **mir-19a** 92095 13q31.3 |
| **mir-23a** 90090 19p13.13 |
| **mir-142** 65837 17q22 |
| **mir-425** 58591 3p21.31 |
| **mir-451** 56728 17q11.2 |
| **mir-140** 44536 16q22.1 |
| **mir-29a** 31210 7q32.3 |
| **mir-27a** 31040 19p13.13 |
| **mir-484** 30789 16p13.11 |
| **mir-301a** 29159 17q22 |
| **let-7g** 27571 3p21.1 |
| **mir-15b** 26955 3q25.33 |
| **mir-23b** 21840 9q22.32 |
| **mir-29c** 21183 1q32.2 |
| **mir-125a** 18815 19q13.41 |
| **mir-107** 15176 10q23.31 |
| **mir-130a** 14145 11q12.1 |
| **mir-30e** 12263 1p34.2 |
| **mir-30b** 10583 8q24.22 |
| **mir-30d** 10304 8q24.22 |
