# Supplementary material for: High-Throughput Sequencing of microRNAs in Peripheral Blood Mononuclear Cells: Identification of Potential Weight Loss Biomarkers
Source: PLoS One. 2013 Jan 15;8(1):e54319. doi: 10.1371/journal.pone.0054319 (PMC3545952; doi:10.1371/journal.pone.0054319)
Supplement: Table S5 — List of predicted novel miRNAs (according to miRBase) found in peripheral blood mononuclear cells. (DOC) [file pone.0054319.s005.doc]

**Supplementary table 5.** List of predicted novel miRNAs (according to miRBase) found in peripheral blood mononuclear cells. The fold change between responders and non-responders is indicated. Student's t-test was used to compare both groups.

| **Name** | **Genomic location** | **Most abundant mature sequence (5p/3p)** | **Fold change**  **Mean** | **P-value**  **Mean** |
| --- | --- | --- | --- | --- |
| novel 1 | chr 19:50295100-50295209_+ | GCCCUGCAUGGUGUCCCCACA | -3.14 | 0.32 |
| novel 2 | chr16:88535323-88535432_+ | GCCAACCGUCAGAGCCCAGAC | -2.10 | 0.23 |
| novel 3 | chr16:29816013-29816124_+ | GCCCAAAGCACUUGGCUGCCCUG | -2.35 | 0.35 |
| novel 4 | chr11:64507262-64507372_- | GGCCACUCCUCAUGCCCCCAG | -2.31 | 0.09 |
| novel 5 | chr10:120819507-120819616_- | GCUGCCAGUCUCCUUCAGAC | -2.59 | 0.45 |
| novel 7 | chr6:133138407-133138519_+ | AGCCAUGUUACGAGCCUUAAGGA | -2.77 | 0.21 |
| novel 8 | chr4:147329729-147329840_+ | ACAAUUGUUGAUCUUGGGCCUG | -6.78 | 0.13 |
| novel 9 | chr3:122692516-122692516_- | CAGAUUCCCAGAGUGGGACAGG | -3.40 | 0.36 |
| novel 10 | chr2:219206612-219206723_+ | CCCCUGCUCCCUUGUUCCCCAG | -4.24 | 0.19 |
| novel 11 | chr2:219144818-219144940_- | UGGGAUUGACGCCACAUGUC | -3.37 | 0.21 |
| novel 12 | chr2:16612710-16612819_- | AUCCUCACUUUGAAUCCAUG | -3.91 | 0.23 |
| novel 13 | chr1:226793200-226793309_+ | CAUGUUUAGUGAUACAAUC | -2.23 | 0.63 |
| novel 14 | chr1:55784327-55784436_+ | GCCUCCCAGUCUGGCCUGAG | -6.35 | 0.15 |
