# Supplementary material for: High-Throughput Sequencing of microRNAs in Peripheral Blood Mononuclear Cells: Identification of Potential Weight Loss Biomarkers
Source: PLoS One. 2013 Jan 15;8(1):e54319. doi: 10.1371/journal.pone.0054319 (PMC3545952; doi:10.1371/journal.pone.0054319)
Supplement: Table S6 — Target mRNAs of the microRNAs with differences in expression between responders and non-responders to the energy-restricted. (DOC) [file pone.0054319.s006.doc]

**Supplementary table 6**. Target mRNAs of the microRNAs with differences in expression between responders and non-responders to the energy-restricted

|  |  |  | **miRanda algorithm** | | | | | **TargetScan algorithm** | |
| --- | --- | --- | --- | --- | --- | --- | --- | --- | --- |
|  |  |  | **MicroCosm** | | | **MicroRNA** | | **TargetScan** | |
|  | ***Target Gene*** | ***Gene Name*** | ***Number of predicted targets*** | ***Score*** | ***P-value*** | ***Number of predicted targets*** | ***mirSVR score*** | ***Number of predicted targets**** | ***Total context+ score*** |
| **mir-935** |  |  | 941 |  |  | 6131 |  | 276 |  |
|  | HDAC1 | Histone deacetylase 1 |  | 15.02 | 0.006 |  |  |  |  |
|  | ADRA1A | Alpha-1A adrenergic receptor |  | 15.66 | <0.001 |  |  |  |  |
|  | ADFP | Adipophilin |  | 15.84 | 0.035 |  |  |  |  |
|  | FFAR3 | Free fatty acid receptor 3 |  | 17.45 | 0.007 |  |  |  |  |
|  | FADS6 | Fatty acid desaturase domain family, member 6 |  | 15.56 | 0.046 |  |  |  |  |
|  | LPL | Lipoprotein lipase precursor |  | 15.25 | 0.009 |  |  |  |  |
|  | RBP5 | Retinol-binding protein III, cellular |  | 15.64 | 0.042 |  |  |  |  |
|  | PKIB | cAMP-dependent protein kinase inhibitor beta |  | 15.90 | 0.033 |  |  |  |  |
|  | APOOL | Apolipoprotein O-like precursor |  | 17.31 | <0.001 |  |  |  |  |
|  | [ADRB1](http://www.ncbi.nlm.nih.gov/sites/entrez?Db=gene&Cmd=ShowDetailView&TermToSearch=153) | Beta-1 adrenergic receptor |  |  |  |  |  |  | -0.24 |
|  | [ACSL3](http://www.ncbi.nlm.nih.gov/sites/entrez?Db=gene&Cmd=ShowDetailView&TermToSearch=2181) | Acyl-CoA synthetase long-chain family member 3 |  |  |  |  |  |  | -0.11 |
|  | [PKIA](http://www.ncbi.nlm.nih.gov/sites/entrez?Db=gene&Cmd=ShowDetailView&TermToSearch=5569) | Protein kinase (cAMP-dependent, catalytic) inhibitor alpha |  |  |  |  |  |  | -0.16 |
|  | HIF1A | Hypoxia-inducible factor 1 alpha |  |  |  |  | -1.47 |  |  |
| **mir-223** |  |  | 999 |  |  | 5449 |  | 311 |  |
|  | PCK2 | Phosphoenolpyruvate carboxylase |  | 15.80 | <0.001 |  |  |  |  |
|  | SIGLEC5 | Obesity- binding protein 2 |  | 15.58 | 0.015 |  |  |  |  |
|  | SLC27A2 | Very-long-chain acyl-CoA synthetase |  | 17.40 | 0.003 |  |  |  |  |
|  | IGFL2 | Insulin growth factor-like family member 2 precursor |  | 16.49 | 0.003 |  |  |  |  |
|  | DNMT1 | DNA (cytosine-5)-methyltransferase 1 |  | 15.69 | 0.008 |  |  |  |  |
|  | HDAC2 | Histone deacetylase 2 |  | 15.69 | <0.001 |  |  |  |  |
|  | HDAC8 | Histone deacetylase 8 |  | 18.31 | 0.004 |  |  |  |  |
|  | DAGLB | Sn1-specific diacylglycerol lipase beta |  | 15.30 | 0.009 |  |  |  |  |
|  | PNLIP | Pancreatic triacylglycerol lipase precursor |  | 16.49 | 0.026 |  |  |  |  |
|  | CCRL2 | C-C chemokine receptor-like 2 |  | 15.39 | <0.001 |  |  |  |  |
|  | SIRT5 | NAD-dependent deacetylase sirtuin-5 |  | 15.23 | 0.001 |  |  |  |  |
|  | [IGF1R](http://www.ncbi.nlm.nih.gov/sites/entrez?Db=gene&Cmd=ShowDetailView&TermToSearch=3480) | Insulin-like growth factor 1 receptor |  |  |  |  |  |  | -0.27 |
|  | [HDAC4](http://www.ncbi.nlm.nih.gov/sites/entrez?Db=gene&Cmd=ShowDetailView&TermToSearch=9759) | Histone deacetylase 4 |  |  |  |  |  |  | >-0.03 |
|  | LIPG | Factor 1, alpha subunit; lipase |  |  |  |  | -1.89 |  |  |
| **mir-224** |  |  | 999 |  |  | 8059 |  | 382 |  |
|  | ELOVL5 | Elongation of very long chain fatty acids protein 5 |  | 16.12 | <0.001 |  |  |  |  |
|  | ELOVL4 | Elongation of very long chain fatty acids protein 4 |  | 16.27 | 0.002 |  |  |  |  |
|  | FABP7 | Fatty acid-binding protein, brain |  | 16.69 | 0.027 |  |  |  |  |
|  | ACOX3 | Acyl-coenzyme A oxidase 3, peroxisomal |  | 17.83 | 0.009 |  |  |  |  |
|  | MC3R | Melanocortin receptor 3 |  | 17.31 | 0.015 |  |  |  |  |
|  | APOL2 | Apolipoprotein-L2 |  | 16.63 | 0.008 |  |  |  |  |
|  | APOL3 | Apolipoprotein-L3 |  | 16.81 | 0.024 |  |  |  |  |
|  | CXCR6 | C-X-C chemokine receptor type |  | 16.59 | <0.001 |  |  |  |  |
|  | VEGFC | Vascular endothelial growth factor C precursor |  | 15.78 | 0.002 |  |  |  |  |
|  | VEGFB | Vascular endothelial growth factor B precursor |  | 16.39 | 0.036 |  |  |  |  |
|  | SREBF1 | Sterol regulatory element-binding protein 1 |  | 16.03 | 0.012 |  |  |  |  |
|  | [IRS2](http://www.ncbi.nlm.nih.gov/sites/entrez?Db=gene&Cmd=ShowDetailView&TermToSearch=8660) | Insulin receptor substrate 2 |  |  |  |  |  |  | -0.10 |
|  | [ACSL4](http://www.ncbi.nlm.nih.gov/sites/entrez?Db=gene&Cmd=ShowDetailView&TermToSearch=2182) | Acyl-CoA synthetase long-chain family member 4 |  |  |  |  |  |  | -0.41 |
|  | [APOOL](http://www.ncbi.nlm.nih.gov/sites/entrez?Db=gene&Cmd=ShowDetailView&TermToSearch=139322) | Apolipoprotein O-like |  |  |  |  |  |  | -0.23 |
|  | ACAT1 | Acetyl-coa acetyltransferase |  |  |  |  | -1.25 |  |  |
| **mir-376b** |  |  | 1063 |  |  | 5542 |  | 212 |  |
|  | ACSL6 | Long-chain acyl-CoA synthetase 6 |  | 17.11 | 0.004 |  |  |  |  |
|  | FABP4 | Fatty acid-binding protein, adipocyte |  | 15.69 | 0.024 |  |  |  |  |
|  | HAT1 | Histone acetyltransferase type B catalytic subunit |  | 15.62 | <0.001 |  |  |  |  |
|  | APOO | Apolipoprotein O precursor |  | 17.85 | 0.008 |  |  |  |  |
|  | CPT2 | Carnitine O-palmitoyltransferase 2, mitochondrial precursor |  | 15.87 | 0.012 |  |  |  |  |
|  | CCL4L1 | Chemokine (C-C motif) ligand 4-like 1 precursor |  | 16.10 | 0.002 |  |  |  |  |
|  | CCRL1 | C-C chemokine receptor type 11 |  | 17.53 | 0.007 |  |  |  |  |
|  | CCL4 | Small inducible cytokine A4 precursor |  | 15.86 | <0.001 |  |  |  |  |
|  | CCL24 | Small inducible cytokine A24 precursor |  | 15.99 | 0.002 |  |  |  |  |
|  | [ACSL1](http://www.ncbi.nlm.nih.gov/sites/entrez?Db=gene&Cmd=ShowDetailView&TermToSearch=2180) | Acyl-CoA synthetase long-chain family member 1 |  |  |  |  |  |  | -0.33 |
|  | [HDAC9](http://www.ncbi.nlm.nih.gov/sites/entrez?Db=gene&Cmd=ShowDetailView&TermToSearch=9734) | Histone deacetylase 9 |  |  |  |  |  |  | -0.32 |
|  | [IGF1R](http://www.ncbi.nlm.nih.gov/sites/entrez?Db=gene&Cmd=ShowDetailView&TermToSearch=3480) | Insulin-like growth factor 1 receptor |  |  |  |  |  |  | -0.16 |
|  | [IL15](http://www.ncbi.nlm.nih.gov/sites/entrez?Db=gene&Cmd=ShowDetailView&TermToSearch=3600) | Interleukin 15 |  |  |  |  |  |  | -0.10 |
|  | LIPH | Lipase h |  |  |  | -2.67 |  |  |  |
|  | PPARG | Peroxisome proliferator-activated receptor gamma |  |  |  | -2.15 |  |  |  |
|  | G6PC2 | Glucose-6-phosphatase, catalytic |  |  |  | -1.84 |  |  |  |
|  | HDAC9 | Histone deacetylase 9 |  |  |  | -1.68 |  |  |  |
|  | NR3C1 | Glucocorticoid receptor |  |  |  | -1.62 |  |  |  |
| **mir-433** |  |  | 1057 |  |  | 6659 |  | 317 |  |
|  | HDAC6 | Histone deacetylase 6 |  | 17.21 | 0.011 |  |  |  |  |
|  | ADRA1A | Alpha-1A adrenergic receptor |  | 17.20 | 0.012 |  |  |  |  |
|  | IGFBP1 | Insulin-like growth factor-binding protein 1 precursor |  | 16.64 | 0.020 |  |  |  |  |
|  | ACOX2 | Acyl-coenzyme A oxidase 2, peroxisomal |  | 15.46 | 0.005 |  |  |  |  |
|  | LPL | Lipoprotein lipase precursor |  | 15.53 | 0.003 |  |  |  |  |
|  | LIPE | Hormone-sensitive lipase |  | 16.60 | 0.021 |  |  |  |  |
|  | CCL20 | Small inducible cytokine A20 precursor |  | 15.24 | 0.006 |  |  |  |  |
|  | CCL18 | Small inducible cytokine A18 precursor |  | 16.67 | 0.019 |  |  |  |  |
|  | [ADRA1A](http://www.ncbi.nlm.nih.gov/sites/entrez?Db=gene&Cmd=ShowDetailView&TermToSearch=148) | Adrenergic, alpha-1A-, receptor |  |  |  |  |  |  | -0.37 |
|  | [CRLF3](http://www.ncbi.nlm.nih.gov/sites/entrez?Db=gene&Cmd=ShowDetailView&TermToSearch=51379) | Cytokine receptor-like factor 3 |  |  |  |  |  |  | -0.19 |
|  | [LEPR](http://www.ncbi.nlm.nih.gov/sites/entrez?Db=gene&Cmd=ShowDetailView&TermToSearch=3953) | Leptin receptor |  |  |  |  |  |  | N/A |
|  | GCLC | Glutamate-cysteine ligase |  |  |  |  | -2.72 |  |  |
|  | FRZB | Frizzled-related protein |  |  |  |  | -1.64 |  |  |
| **mir-154** |  |  | 874 |  |  | 5307 |  | 129 |  |
|  | SIGLEC6 | Obesity- binding protein 1 |  | 16.17 | 0.001 |  |  |  |  |
|  | ELOVL3 | Elongation of very long chain fatty acids protein 3 |  | 16.49 | 0.024 |  |  |  |  |
|  | ADRA1A | Alpha-1A adrenergic receptor |  | 17.96 | 0.005 |  |  |  |  |
|  | PKIB | cAMP-dependent protein kinase inhibitor beta |  | 15.46 | 0.005 |  |  |  |  |
|  | PNPLA3 | Adiponutrine |  | 16.67 | 0.002 |  |  |  |  |
|  | TNFAIP6 | Tumor necrosis factor-inducible protein TSG-6 precursor |  | 16.26 | 0.013 |  |  |  |  |
|  | IGF1 | Insulin-like growth factor IA precursor (IGF-IA) |  | 17.29 | 0.010 |  |  |  |  |
|  | VEGFC | Vascular endothelial growth factor C precursor growth factor-related protein |  | 16.69 | 0.020 |  |  |  |  |
|  | VEGFB | Vascular endothelial growth factor B precursor |  | 15.31 | 0.020 |  |  |  |  |
|  | IL20 | Interleukin-20 precursor |  | 16.50 | <0.001 |  |  |  |  |
|  | [TNFAIP3](http://www.ncbi.nlm.nih.gov/sites/entrez?Db=gene&Cmd=ShowDetailView&TermToSearch=7128) | Tumor necrosis factor, alpha-induced protein 3 |  |  |  |  |  |  | -0.16 |
| **mir-27b** |  |  | 1074 |  |  | 9156 |  | 1211 |  |
|  | ELOVL1 | Elongation of very long chain fatty acids protein 1 |  | 15.96 | 0.008 |  |  |  |  |
|  | PPARG | Peroxisome proliferator-activated receptor gamma |  | 17.37 | 0.021 |  |  |  |  |
|  | ISL1 | Insulin gene enhancer protein ISL-1 |  | 15.71 | <0.001 |  |  |  |  |
|  | INSR | Insulin receptor precursor |  | 17.41 | <0.001 |  |  |  |  |
|  | MC4R | Melanocortin receptor 4 |  | 16.08 | 0.004 |  |  |  |  |
|  | VEGFC | Vascular endothelial growth factor C precursor (VEGF-C) |  | 16.55 | <0.001 |  |  |  |  |
|  | SIRT5 | NAD-dependent deacetylase sirtuin-5 |  | 17.43 | 0.020 |  |  |  |  |
|  | IL10RB | Interleukin-10 receptor beta chain precursor |  | 16.23 | 0.004 |  |  |  |  |
|  | [PPARG](http://www.ncbi.nlm.nih.gov/sites/entrez?Db=gene&Cmd=ShowDetailView&TermToSearch=5468) | Peroxisome proliferator-activated receptor gamma |  |  |  |  |  |  | -0.41 |
|  | [INSR](http://www.ncbi.nlm.nih.gov/sites/entrez?Db=gene&Cmd=ShowDetailView&TermToSearch=3643) | Insulin receptor |  |  |  |  |  |  | -0.28 |
|  | [IRS1](http://www.ncbi.nlm.nih.gov/sites/entrez?Db=gene&Cmd=ShowDetailView&TermToSearch=3667) | Insulin receptor substrate 1 |  |  |  |  |  |  | -0.16 |
|  | [CNR1](http://www.ncbi.nlm.nih.gov/sites/entrez?Db=gene&Cmd=ShowDetailView&TermToSearch=1268) | Cannabinoid receptor 1 (brain) |  |  |  |  |  |  | -0.05 |
|  | [PPARGC1B](http://www.ncbi.nlm.nih.gov/sites/entrez?Db=gene&Cmd=ShowDetailView&TermToSearch=133522) | Peroxisome proliferator-activated receptor gamma, coactivator 1 beta |  |  |  |  |  |  | >-0.04 |
|  | [IGF1](http://www.ncbi.nlm.nih.gov/sites/entrez?Db=gene&Cmd=ShowDetailView&TermToSearch=3479) | Insulin-like growth factor 1 |  |  |  |  |  |  | >-0.02 |
|  | [LPIN2](http://www.ncbi.nlm.nih.gov/sites/entrez?Db=gene&Cmd=ShowDetailView&TermToSearch=9663) | Lipin 2 |  |  |  |  |  |  | -0.02 |
| **mir-409** |  |  |  |  |  |  |  |  |  |
|  | FZD3 | Frizzled, drosophila, homolog of |  |  |  | 3378 | -1.49 |  |  |
|  | PPARGC1A | Peroxisome proliferator-activated receptor gamma, coactivator 1 alpha |  |  |  |  | -1.30 |  |  |
|  | APOF | Apolipoprotein F |  |  |  |  | -1.24 |  |  |
|  | AGPAT9 | 1-acylglycerol-3-phosphate o-acyltransferase |  |  |  |  | -1.16 |  |  |
| **mir-183** |  |  | 1100 |  |  | 7495 |  | 387 |  |
|  | LRP6 | Low density lipoprotein receptor-related protein 6 |  |  |  |  |  |  | -0.47 |
|  | GPR34 | G protein-coupled receptor 34 |  |  |  |  |  |  | -0.47 |
|  | NPAS3 | Neuronal PAS domain protein 3 |  |  |  |  |  |  | -0.44 |
|  | IL1RL1 | Interleukin 1 receptor-like 1 |  |  |  |  |  |  | -0.42 |
|  | PEX19 | peroxisomal biogenesis factor 19 |  |  |  |  |  |  | -0.42 |
|  | HDAC10 | Histone deacetylase 10 |  | 18.19 | 0.005 |  |  |  |  |
|  | HDAC8 | Histone deacetylase 8 |  | 15.25 | 0.008 |  |  |  |  |
|  | HDAC6 | Histone deacetylase 6 |  | 15.92 | 0.049 |  |  |  |  |
|  | LRP6 | Low-density lipoprotein receptor-related protein 6 precursor. |  | 18.49 | <0.001 |  |  |  |  |
|  | APOA1BP | Apolipoprotein A-I binding protein precursor |  | 16.15 | <0.001 |  |  |  |  |
|  | VLDLR | Very low-density lipoprotein receptor precursor |  | 15.26 | 0.016 |  |  |  |  |
|  | CRTC2 | Transducer of regulated cAMP response element-binding protein (CREB) 2 |  | 16.20 | 0.038 |  |  |  |  |
|  | MAP3K4 | Mitogen-activated protein kinase kinase kinase 4 |  | 16.50 | 0.028 |  |  |  |  |
|  | DGKH | Diacylglycerol kinase eta |  | 15.28 | 0.001 |  |  |  |  |
|  | GK5 | Glycerol kinase 5 |  | 16.25 | 0.001 |  |  |  |  |
|  | PLB1 | Phospholipase B1 |  | 16.21 | 0.037 |  |  |  |  |
|  | FABP7 | Fatty acid-binding protein, brain |  | 16.49 | 0.029 |  |  |  |  |
|  | FIGF | Vascular endothelial growth factor D precursor |  | 15.37 | <0.001 |  |  |  |  |
|  | CPT2 | Carnitine O-palmitoyltransferase 2, mitochondrial precursor |  | 15.24 | 0.009 |  |  |  |  |
|  | ACAD8 | Acyl-CoA dehydrogenase family member 8 |  |  |  |  | -0.06 |  |  |
|  | SCP2 | Sterol carrier protein 2 |  |  |  |  | -0.78 |  |  |
|  | CD36 | CD36 molecule (thrombospondin receptor) |  |  |  |  | -0.64 |  |  |
| **mir-542-3p** |  |  | 993 |  |  | 7129 |  | 279 |  |
|  | IL33 | Interleukin 33 |  |  |  |  |  |  | -0.47 |
|  | GDPD4 | Glycerophosphodiester phosphodiesterase domain containing 4 |  |  |  |  |  |  | -0.47 |
|  | GNB5 | Guanine nucleotide binding protein (G protein), beta 5 |  |  |  |  |  |  | -0.46 |
|  | PLA2R1 | Phospholipase A2 receptor 1 |  |  |  |  |  |  | -0.49 |
|  | IGFBP1 | Insulin-like growth factor-binding protein 1 precursor |  | 16.75 | 0.002 |  |  |  |  |
|  | IGFBP6 | Insulin-like growth factor-binding protein 6 precurso |  | 15.81 | 0.005 |  |  |  |  |
|  | FABP3 | Fatty acid-binding proteín 3 |  | 18.61 | 0.006 |  |  |  |  |
|  | PLA2G4B | Cytosolic phospholipase A2 beta |  | 16.60 | 0.005 |  |  |  |  |
|  | LIPA | Lysosomal acid lipase/cholesteryl ester hydrolase precursor |  | 17.33 | 0.005 |  |  |  |  |
|  | APOL5 | Apolipoprotein-L5 |  | 18.08 | 0.009 |  |  |  |  |
|  | ELOVL3 | Elongation of very long chain fatty acids protein 3 |  | 15.82 | 0.003 |  |  |  |  |
|  | SLC27A3 | Long-chain fatty acid transport protein 3 |  | 15.58 | 0.009 |  |  |  |  |
|  | PPARD | Peroxisome proliferator-activated receptor delta |  | 16.41 | 0.043 |  |  |  |  |
|  | TNF | Tumor necrosis factor precursor |  | 17.06 | 0.001 |  |  |  |  |
|  | FABP3 | Fatty acid-binding proteín 3 |  |  |  |  | -1.45 |  |  |
|  | ACAT1 | Acetyl-CoA acetyltransferase 1 |  |  |  |  | -1.23 |  |  |
|  |  |  |  |  |  |  |  |  |  |
| **mir-4772-3p** |  |  |  |  |  |  |  |  |  |
|  |  |  |  |  |  |  |  | 265 |  |
|  | ACSL1 | Acyl-CoA synthetase long-chain family member 1 |  |  |  |  |  |  | -0.34 |
|  | PDK3 | Pyruvate dehydrogenase kinase, isozyme 3 |  |  |  |  |  |  | -0.33 |
|  | MAP4K5 | Mitogen-activated protein kinase kinase kinase kinase 5 |  |  |  |  |  |  | -0.21 |
|  | NFAT5 | Nuclear factor of activated T-cells 5, tonicity-responsive |  |  |  |  |  |  | -0.21 |
|  | PPARGC1A | Peroxisome proliferator-activated receptor gamma, coactivator 1 alpha |  |  |  |  |  |  | -0.06 |
|  | ERLIN2 | ER lipid raft associated 2 |  |  |  |  |  |  | -0.06 |
|  | IGF2BP1 | Insulin-like growth factor 2 mRNA binding protein 1 |  |  |  |  |  |  | > -0.02 |
|  | IGF2 | Insulin-like growth factor 2 (somatomedin A) |  |  |  |  |  |  | > -0.01 |
|  | LRP8 | Low density lipoprotein receptor-related protein 8, apolipoprotein e receptor |  |  |  |  |  |  | > -0.01 |
|  | MAP3K3 | Mitogen-activated protein kinase kinase kinase 3 |  |  |  |  |  |  | > -0.01 |
|  | CREBBP | CREB binding protein |  |  |  |  |  |  | -0.07 |
|  |  |  |  |  |  |  |  |  |  |
|  |  |  |  |  |  |  |  |  |  |
